# ENPP1’s regulation of extracellular cGAMP is a ubiquitous mechanism of attenuating STING signaling

**DOI:** 10.1073/pnas.2119189119

**Published:** 2022-05-19

**Authors:** Jacqueline A. Carozza, Anthony F. Cordova, Jenifer A. Brown, Yasmeen AlSaif, Volker Böhnert, Xujun Cao, Rachel E. Mardjuki, Gemini Skariah, Daniel Fernandez, Lingyin Li

**Affiliations:** ^a^ChEM-H Institute, Stanford University, Stanford, CA 94301;; ^b^Department of Chemistry, Stanford University, Stanford, CA 94301;; ^c^Cancer Biology Program, Stanford University, Stanford, CA 94301;; ^d^Department of Biophysics, Stanford University, Stanford, CA 94301;; ^e^Department of Biology, Stanford University, Stanford, CA 94301;; ^f^Department of Biochemistry, Stanford University, Stanford, CA 94301;; ^g^Macromolecular Structural Knowledge Center, Stanford University, Stanford, CA 94301

**Keywords:** cGAMP, ENPP1, STING, extracellular cGAMP, immunotransmitter

## Abstract

The metazoan innate immune second messenger 2′3′-cGAMP is present both inside and outside cells. However, only extracellular cGAMP can be negatively regulated by the extracellular hydrolase ENPP1. Here, we determine whether ENPP1’s regulation of extracellular cGAMP is a ubiquitous mechanism of attenuating stimulator of interferon genes (STING) signaling. We identified ENPP1^H362A^, a point mutation that cannot degrade the 2′-5′ linkage in cGAMP while maintaining otherwise normal function. The selectivity of this histidine is conserved down to bacterial nucleotide pyrophosphatase/phosphodiesterase (NPP), allowing structural analysis and suggesting an unexplored ancient history of 2′-5′ cyclic dinucleotides. *Enpp1^H362A^* mice demonstrated that extracellular cGAMP is not responsible for the devastating phenotype in ENPP1-null humans and mice but is responsible for antiviral immunity and systemic inflammation. Our data define extracellular cGAMP as a pivotal STING activator, identify an evolutionarily critical role for ENPP1 in regulating inflammation, and suggest a therapeutic strategy for viral and inflammatory conditions by manipulating ENPP1 activity.

## Significance

The immune system strikes a careful balance between launching a robust response to threats and avoiding overactivation. The molecule cGAMP is an immunotransmitter that activates innate immunity and signals extracellularly, where it is subject to degradation by the enzyme ENPP1. Here, we engineer ENPP1 to lose activity toward cGAMP but not other substrates, thus creating a biochemically precise tool to understand how ENPP1 regulates extracellular cGAMP and thus innate immunity. We uncover that ENPP1's degradation of extracellular cGAMP has a long evolutionary history, and that this mechanism is critical for controlling diverse immune threats, including viral infection and inflammation.

Since its discovery 9 y ago, 2′3′-cyclic-GMP-AMP (cGAMP) quickly became the intense focus of many biochemistry, immunology, and cell biology laboratories (1–3). Its significance was immediately apparent, being a new addition to the relatively small group of known second messengers. cGAMP is synthesized by the enzyme cyclic-GMP-AMP synthase (cGAS) in response to self and pathogenic double-stranded DNA in the cytosol. cGAMP then binds its endoplasmic reticulum membrane-localized receptor stimulator of interferon genes (STING) to activate downstream transcription of interferon (IFN) and other cytokines, triggering powerful antiviral (1, 4) and anticancer (5–9) defense mechanisms. However, it also triggers devastating inflammation in neurodegenerative diseases (10, 11), myocardial infarction (12), and autoimmune diseases (13–16) when not properly regulated.

cGAMP’s regulation depends on its cellular localization. cGAMP was originally thought to function as an intracellular signal that is synthesized by cGAS and sensed by STING within the same cell. cGAMP was subsequently found to spread to neighboring cells through gap junctions (17–19) and exosomes (20, 21), both of which do not permit access to the extracellular space. The prevailing view that cGAMP is exclusively an intracellular signal was challenged by the recent discovery that cGAMP is secreted into the extracellular space by cancer cells and functions as a paracrine immunotransmitter by entering and activating host immune cells (19, 22–26). Intracellular cGAMP signaling is primarily regulated at the level of STING activation, which requires the formation of a large signaling complex (27–29) that has a high activation threshold but is irreversible once formed (27), explaining its role in uncontrolled inflammation. Extracellular cGAMP signaling is controlled by many additional molecular and cellular mechanisms. cGAMP is transported by cell-type–specific transporters (25, 26, 30–33), allowing for cell-specific secretion and uptake. Additionally, extracellular cGAMP is degraded by the extracellular hydrolase ENPP1, while no intracellular cGAMP hydrolase has been reported (22, 34).

The studies that established cancer-to-host cGAMP immunotransmission were enabled by experimental techniques that are only possible in cancer models, including genetic manipulation of cancer cells (19, 22, 23, 26) and local extracellular cGAMP depletion through intratumoral injections of a depleting agent (22, 25). ENPP1 was subsequently shown to be a negative regulator of anticancer innate immunity, enabling the development of ENPP1 inhibitors (patents WO2020160333A1, WO2019023635A1, WO2018119328A1) as investigative new drugs for cancer therapy. Whether ENPP1-regulated extracellular cGAMP signaling is a ubiquitous mechanism of STING signaling remains a major unsolved question, with the potential to impact therapies targeting ENPP1 in broad disease contexts.

## Results

### Mutations of Guanosine-Adjacent Residues in ENPP1 Do Not Inhibit cGAMP Hydrolysis.

To understand the role that extracellular cGAMP plays as an immunotransmitter in diseases beyond cancer, we sought to develop a universal tool that can systemically manipulate extracellular cGAMP levels. We hypothesized that the cGAMP hydrolase ENPP1 was a promising candidate to build such a tool, since ENPP1 degrades extracellular but not intracellular cGAMP (22). However, ENPP1 also degrades extracellular ATP to AMP and pyrophosphate (PP_i_), the latter of which is critical in regulating calcium homeostasis (35). Organisms lacking ENPP1 exhibit severe systemic calcification and a significantly shortened lifespan (36–38). In addition, extracellular ATP and its downstream degradation products AMP and adenosine are all immunomodulatory molecules. Therefore, we sought to identify substrate-selective ENPP1 mutations to specifically study the role of extracellular cGAMP.

**Fig. 1.**
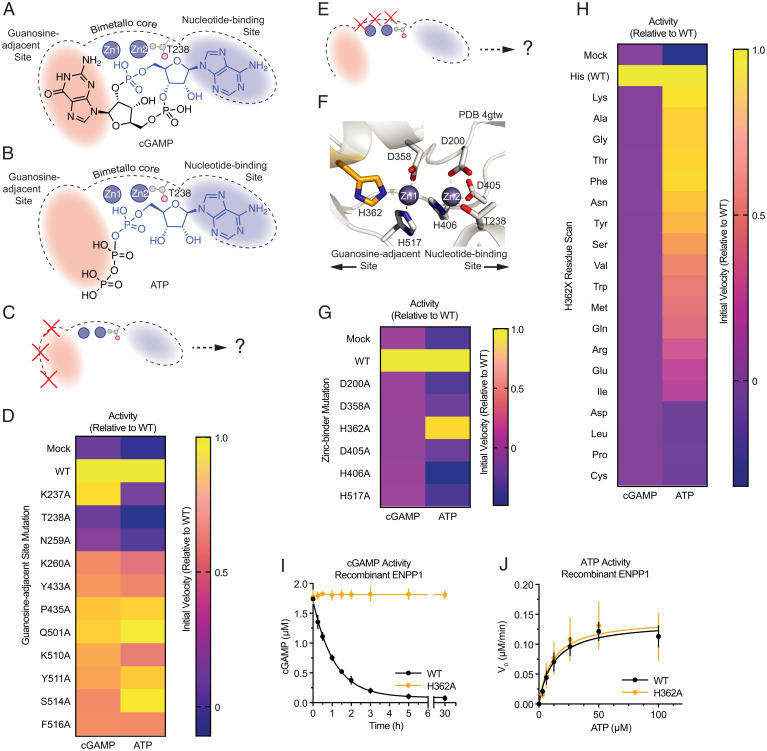
Discovery and characterization of ENPP1^H362A^, a mutation that degrades ATP but not cGAMP. (*A* and *B*) Schematic illustration of the ENPP1 active site bound to substrates cGAMP (*A*) and ATP (*B*). (*C*) Schematic illustration of proposed mutations (red Xs) of the residues in the guanosine-adjacent site (red). (*D*) Heat map showing the initial velocity relative to WT of guanosine-adjacent residue mutations for the substrates cGAMP and ATP at pH 9. The mean initial velocity was calculated from a linear fit of the degradation reactions during early time points; *n* = 4 independent reactions. (*E*) Schematic illustration of proposed mutations (red Xs) of the zinc-binding residues (zincs shown as dark gray spheres). (*F*) The catalytic center of mouse ENPP1 (PDB ID code: 4GTW). Zinc-binding residues shown as gray (D200, T238, D358, D405, H406, and H517) or orange (H362) sticks; zincs shown as dark gray spheres. (*G*) Heat map showing the initial velocity relative to WT of zinc-binding residues for the substrates cGAMP and ATP at pH 9. The mean initial velocity was calculated from a linear fit of the degradation reactions during early time points; *n* = 3 independent reactions. (*H*) Heat map showing the initial velocity relative to WT of H362X mutations (where X is each amino acid), for the substrates cGAMP and ATP at pH 9. The mean initial velocity was calculated from a linear fit of the degradation reactions during early time points; *n* = 3 independent reactions. (*I*) Kinetic analysis of cGAMP activity monitoring degradation products by thin-layer chromatography (TLC) using recombinant purified ENPP1; *n* = 3 independent reactions, mean ± SD with some error bars too small to visualize. (*J*) Michaelis–Menten plots of ATP activity monitoring AMP production using recombinant purified ENPP1; *n* = 3 independent reactions, mean ± SEM. WT: *K*_m_ = 12.1 µM, *k*_cat_ = 0.76 s^−1^, *k*_cat_/*K*_m_ = 6.3 × 10^4^ M^−1^s^−1^. H362A: *K*_m_ = 11.1 µM, *k*_cat_ = 0.79 s^−1^, *k*_cat_/*K*_m_ = 7.1 × 10^4^ M^−1^s^−1^.

We first compared the cocrystal structures of mouse ENPP1 bound to AMP (39) and pApG, the intermediate after the first phosphodiester bond cleavage of cGAMP (40). ENPP1 has a tight nucleotide-binding site, which the AMP portions of ATP and cGAMP occupy, and a secondary site, which the guanine base of cGAMP occupies (guanosine-adjacent site) (Fig. 1 *A* and *B*). Between the two sites, a cluster of residues chelate two zinc ions, which position the α-phosphate of the substrates for nucleophilic attack by the catalytic residue T238. We validated our previously developed enzymatic assay using ENPP1^WT^ and ENPP1^T238A^ (*SI Appendix*, Fig. S1*A*) as positive and negative controls for both substrates. We generated whole-cell lysates by overexpressing FLAG-tagged ENPP1 variants in 293T *ENPP1^−/−^* cells (*SI Appendix*, Fig. S1*B*) as a fast and robust source of mutant enzymes. [^32^P]-cGAMP degradation was monitored using thin-layer chromatography separation and autoradiography, and ATP degradation was monitored using a luciferase-based assay. Using these assays, we confirmed that ENPP1^WT^ is able to degrade both ATP and cGAMP, while ENPP1^T238A^ is not (*SI Appendix*, Fig. S1 *C* and *D*).

Inactivating the nucleotide-binding site is known to prevent both cGAMP (41) and ATP hydrolysis (39) (*SI Appendix*, Fig. S1*E*). Therefore, we sought to identify residues that are required for cGAMP but not ATP hydrolysis by individually mutating 10 residues with sidechains within 5 Å of the guanine base of cGAMP (Fig. 1*C* and *SI Appendix*, Fig. S1 *F* and *G*) and measuring their initial rates of substrate hydrolysis (Fig. 1*D* and *SI Appendix*, Fig. S1 *H*–*R*). Surprisingly, most of these mutations had only a modest effect on cGAMP degradation. ATP activity usually tracked with cGAMP activity, suggesting that these mutations led to general destabilization. Mutation of N259, a highly conserved residue that forms a hydrogen bond with the nonbridging phosphoryl oxygen of the substrates (42, 43), inhibited activity for both substrates. The K237A mutation achieved the opposite of our goal by abolishing ATP degradation but preserving cGAMP degradation. It is possible that K237 is important for stabilizing the β- and γ-phosphates of ATP (44), but is too far away from the phosphodiester of cGAMP to affect catalysis.

### Discovery and Characterization of ENPP1^H362A^, a Mutation That Degrades ATP but Not cGAMP.

We next made alanine mutations of the six aspartate and histidine zinc-binding residues that comprise the catalytic core of ENPP1 (Fig. 1 *E* and *F* and *SI Appendix*, Fig. S2*A*). All of these mutations were inactive toward both substrates except for the H362A mutation (ENPP1^H362A^). Remarkably, this mutation recapitulated ENPP1^WT^ activity toward ATP but had no detectable activity toward cGAMP (Fig. 1*G* and *SI Appendix*, Fig. S2 *B*–*J*). We then mutated H362 to the other 18 amino acids, all of which were inactive toward cGAMP, indicating that histidine is essential at that position for cGAMP degradation (Fig. 1*H* and *SI Appendix*, Fig. S3 *A*–*E*). Finally, the purified ENPP1^H362A^ enzyme also had no detectable cGAMP hydrolysis activity (Fig. 1*I* and *SI Appendix*, Fig. S3*F*) but had identical *k*_cat_/*K*_m_ values to ENPP1^WT^ for ATP hydrolysis (Fig. 1*J*). As expected, ENPP1^H362A^ was also fully active toward the other NTP substrates of ENPP1 (GTP, UTP, and CTP) (*SI Appendix*, Fig. S3*G*).

### Bacterial NPP Selectively Cleaves 2′-5′ Linkages in Cyclic Dinucleotides Using the Conserved Histidine.

**Fig. 2.**
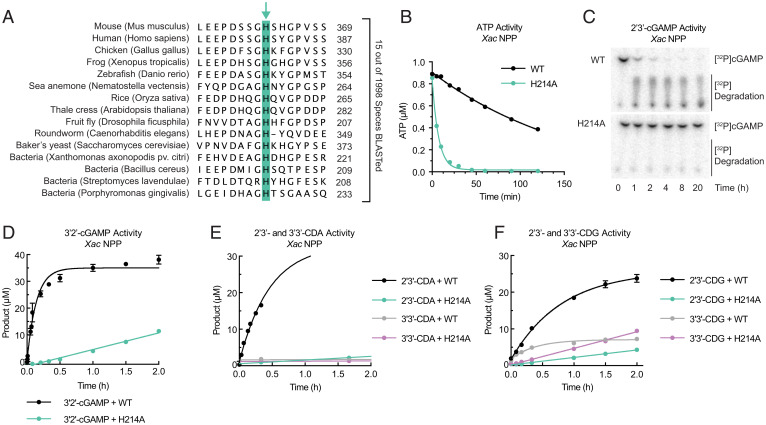
Bacterial NPP selectively cleaves 2′-5′ linkages in cyclic dinucleotides using the conserved histidine. (*A*) Sequence alignment of NPP from a range of species showing the conserved histidine (highlighted in green box). (*B* and *C*) TLC of ATP (*B*) and cGAMP (*C*) degradation by *Xac* NPP^WT^ compared to *Xac* NPP^H214A^ (both at 3 µM enzyme concentration). In *B*, *n* = 3 independent reactions, mean ± SD with some error bars too small to visualize; *C* is representative of three independent reactions from two independent protein purifications. (*D*–*F*) Degradation of 3′2′-cGAMP (*D*), 2′3′-CDA and 3′3′-CDA (*E*), and 2′3′-CDG and 3′3′-CDG (*F*) by *Xac* NPP^WT^ and NPP^H214A^. Product formation was measured by coupling the reaction to 1 U alkaline phosphatase and measuring resultant P_i_; *n* = 3 independent reactions, mean ± SD with some error bars too small to visualize. Data were fit to one-phase decay.

Since ENPP1 is part of the highly conserved nucleotide pyrophosphatase/phosphodiesterase (NPP) protein family, we investigated the evolutionary conservation of the analogous histidine residue and its requirement for cGAMP degradation. This histidine is 100% conserved in the 998 eukaryotic species and 99.9% conserved in the 1,000 bacterial species that we investigated (Fig. 2*A* and *SI Appendix*, Fig. S4*A*). We then selected a small pool of eukaryotic and prokaryotic NPP sequences to investigate their cellular localization. All the sequences we investigated had a predicted signal peptide (*SI Appendix*, Fig. S4*B*), suggesting extracellular localization in eukaryotes or periplasmic localization in bacteria. However, the physiological substrates and the role of NPP in bacteria remain unknown. One hypothesis is that bacterial NPP in the periplasm hydrolyzes nucleotide triphosphates, such as ATP, to salvage the base products (45). We therefore tested the ability of bacterial *Xanthomonas axonopodis* pv*. citri* (*Xac*) NPP (42, 46) to degrade ATP. We found that *Xac* NPP^WT^ degrades ATP and that *Xac* NPP^H214A^ (which corresponds to the mouse H362A mutation) degrades ATP several-fold faster than NPP^WT^ (Fig. 2*B*), suggesting that H214 is not conserved for the purpose of scavenging base products from ATP.

We then tested the hypothesis that the conserved histidine is important for cleaving cyclic dinucleotides. Indeed, *Xac* NPP^WT^ degrades the metazoan 2′3′-cGAMP while the NPP^H214A^ mutant does not, mirroring our findings with mouse ENPP1^H362A^ (Fig. 2*C*). A different 2′-5′–linked cGAMP, 3′2′-cGAMP, was recently discovered as a second messenger in a bacterial antiviral system (47). We found that *Xac* NPP^WT^ readily degrades 3′2′-cGAMP (Fig. 2*D*). In addition, *Xac* NPP^WT^ degrades other synthetic cyclic dinucleotides with 2′-5′/3′-5′ mixed linkages much faster than their counterparts with two 3′-5′ linkages (Fig. 2 *E* and *F* and *SI Appendix*, Fig. S4 *C*–*F*). Interestingly, H214 is required to cleave cyclic dinucleotides with 2′-5′ linkages (2′3′-cGAMP, 3′2′-cGAMP, 2′3′-CDA, 2′3′-CDG), but is not required for cyclic dinucleotides with only 3′-5′ linkages (3′3′-cGAMP, 3′3-CDA, or 3′3-CDG) (Fig. 2 *D*–*F* and *SI Appendix*, Fig. S4 *G*–*I*). This substrate preference and requirement for the remarkably conserved histidine suggest that bacterial NPP’s canonical substrates may be 2′-5′–linked cyclic dinucleotides or oligonucleotides, including the recently discovered 3′2′-cGAMP (48).

### Structure-Guided Elucidation of the Substrate-Selective Degradation Mechanism of ENPP1^H362A^.

**Fig. 3.**
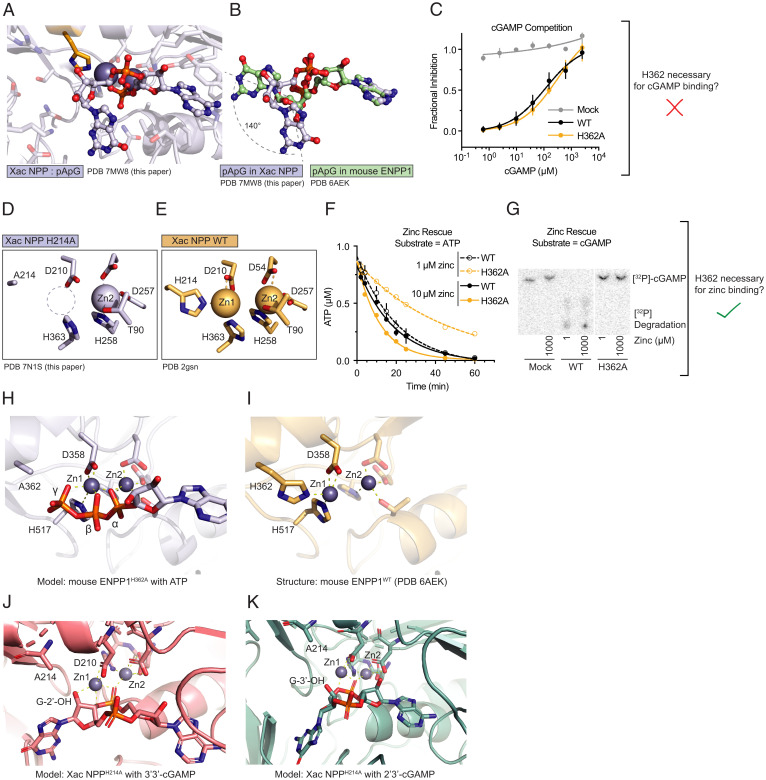
Structure-guided elucidation of the substrate-selective degradation mechanism of ENPP1^H362A^. (*A*) Cocrystal structure of *Xac* NPP^T90A^ (gray cartoon and sticks) with linear cGAMP intermediate pApG (gray spheres and sticks). Zinc atoms are depicted as dark gray spheres. (*B*) Comparison of pApG bound in *Xac* NPP^T90A^ (gray) versus mouse ENPP1^T238A^ (green). (*C*) Competitive inhibition of ATP hydrolysis with cGAMP at pH 7.5; *n* = 3 independent reactions, mean ± SD. WT: *K*_i_ = 120 µM. H362A: *K*_i_ = 320 µM. (*D* and *E*) Crystal structures of *Xac* NPP^H214A^ with one zinc bound (*D*) and *Xac* NPP^WT^ [PDB ID code: 2GSN (42)] with two zincs bound (*E*). (*F* and *G*) Zinc rescue experiments with ATP (*F*) or cGAMP (*G*) as the substrate; *n* = 3 independent reactions. Mean ± SD shown for ATP (*F*), representative reaction shown for cGAMP (*G*). (*H* and *I*) Model of mouse ENPP1^H362A^ (gray cartoon and sticks) with intact ATP (gray sticks and spheres) chelating Zn1 (*H*) compared to mouse ENPP1^WT^ (gold cartoon and sticks) with H362 chelating Zn1 (*I*). (*J* and *K*) Model of *Xac* NPP^H214A^ bound to 3′3′-cGAMP (*J*) and 2′3′-cGAMP (*K*).

We then sought to use structural analysis to explain the mechanism of the exquisite substrate selectivity of *Xac* NPP^H214A^ and mouse ENPP1^H362A^ toward nucleotide triphosphates and 3′-5′ phosphodiester bonds, but not 2′-5′ phosphodiester bonds. As mouse ENPP1 is technically challenging to crystallize, we took advantage of the conservation between *Xac* NPP and mouse ENPP1 to solve the cocrystal structure of *Xac* NPP in complex with cGAMP. To prevent cGAMP hydrolysis, we mutated the catalytic threonine to alanine (*Xac* NPP T90A), crystalized it with cGAMP, and obtained a 1.9 Å crystal structure (*SI Appendix*, Tables S1 and S2). Similar to the previous mouse ENPP1 T238A structure (40), we found a linear 3′-5′–linked pApG intermediate bound to NPP instead of intact cGAMP (Fig. 3*A*). In the previous structure, the guanine ring was within stacking distance of the conserved histidine (∼4.5 Å). However, the guanine ring in our structure is ∼8 Å away from the histidine and rotated 140° from the previous structure (Fig. 3*B*). This discrepancy suggests that the guanine ring is flexible and mobile, which explains why mutating the guanosine-adjacent site did not disrupt cGAMP hydrolysis. Using cGAMP as a competitive inhibitor of the ENPP1-ATP reaction, we determined that ENPP1^WT^ and ENPP1^H362A^ have very similar *K*_i_ values, demonstrating that ENPP1^H362A^ binds cGAMP equally well as ENPP1^WT^ (Fig. 3*C*). Together, our structural and biochemical data demonstrate that H362 is not necessary for binding cGAMP.

Two zinc ions are required for NPP’s catalytic activity, with Zn1 being particularly important for stabilizing the leaving group, whereas Zn2 activates the threonine nucleophile (42, 49). Since the histidine (H362 in mouse ENPP1 and H214 in *Xac* NPP) is one of three residues that chelates Zn1, we hypothesized that the loss of the histidine prevents Zn1 binding. Indeed, in contrast to *Xac* NPP^WT^, which binds two zincs, our 2.0 Å crystal structure revealed that *Xac* NPP^H214A^ was missing Zn1 (Fig. 3 *D* and *E*). We then followed up this structural insight with biochemical experiments using mouse ENPP1. Increasing the zinc concentration from 1 μM to 10 μM sped up the rate of ATP hydrolysis for ENPP1^H362A^, but had no effect on ENPP1^WT^, suggesting that the missing histidine lowers the affinity for Zn1 (Fig. 3*F*). However, we were unable to rescue cGAMP activity even with 1 mM zinc, which is 100-fold above physiological conditions (10 to 20 μM) (Fig. 3*G*).

To explain this discrepancy in how the two substrates are differentially impacted by zinc concentrations, we hypothesize that ATP is able to replace the histidine to chelate Zn1, as ATP in solution is usually chelated to metals (50–52) and binds to zinc with a *K*_d_ of ∼10 to 100 μM, depending on the solution conditions (52). We tested this hypothesis by modeling zinc-bound ATP in mouse ENPP1^H362A^ (Fig. 3*H*). In our model, the α-, β-, and γ-phosphates of ATP all chelated Zn1 in conjunction with H517 and D200. In fact, the γ-phosphate oxygen was in a very similar position as the H362 nitrogen that coordinates Zn1 in ENPP1^WT^ (Fig. 3*I*). We also docked 3′3′-cGAMP and 2′3′-cGAMP into our H214A *Xac* NPP structure. Similar to ATP, the free 2′-OH group in 3′3′-cGAMP can also take the place of the missing histidine to chelate Zn1 while the free 3′-OH group in 2′3′-cGAMP is too far away from Zn1 (Fig. 3 *J* and *K*). We propose that ATP and 3′-5′–linked cyclic dinucleotides are able to compensate for the loss of histidine with their γ-phosphate oxygen and 2′-OH group, respectively, explaining why H362 in mouse ENPP1 and H214 in *Xac* NPP are only required for cleaving 2′-5′–linked phosphodiester bonds.

### *Enpp1^H362A^* Mice Can Degrade ATP but Not cGAMP.

**Fig. 4.**
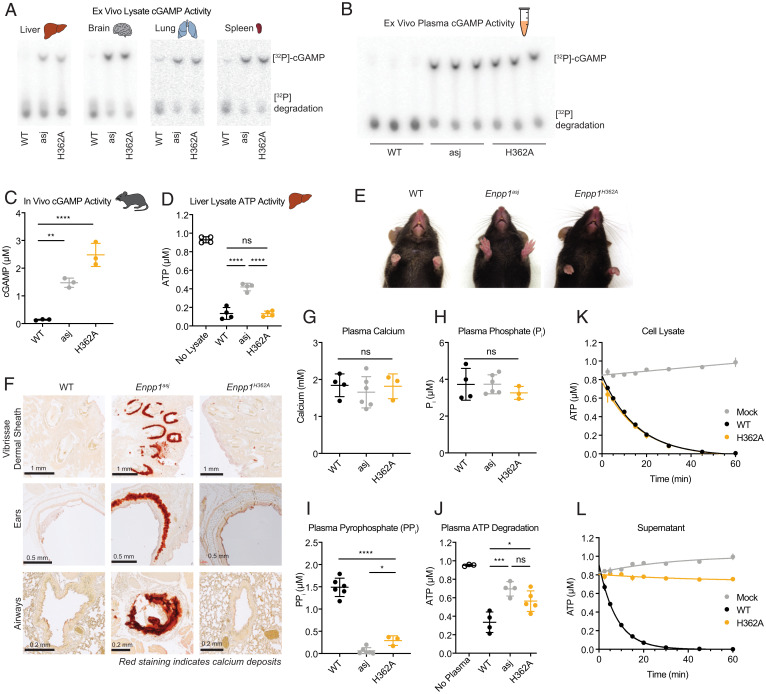
*Enpp1^H362A^* mice do not exhibit the severe systemic calcification seen in ENPP1-null humans and mice. (*A*) Ex vivo organ lysate (1 mg/mL) cGAMP degradation at pH 9 assessed by TLC after 4 h (liver, brain) or 2 h (lung, spleen); *n* = 1 mouse per genotype. (*B*) Ex vivo plasma cGAMP degradation assessed by TLC after 4 h; *n* = 3 mice per genotype. (*C*) In vivo cGAMP degradation: 5 mg/kg of cGAMP was injected subcutaneously into each mouse. Blood was collected from each mouse after 30 min and cGAMP was measured by LC-MS/MS; *n* = 3 mice per genotype. (*D*) Ex vivo liver lysate (1 mg/mL) ATP degradation at pH 7.5 assessed after 20 min; *n* = 4 mice per genotype. (*E*) Photos of *Enpp1^WT^*, *Enpp1^H362A^*, and *Enpp1^asj^* mouse paws. The *Enpp1^asj^* mouse paws are unable to relax due to the calcified joints. (*F*) Twenty-week-old mice were euthanized and the ears, airways, and vibrissae were fixed and stained with Alizarin red to identify calcium deposits. Representative images are shown from one to two mice per genotype. (*G* and *H*) Plasma calcium (*G*) and phosphate (*H*) concentrations in *Enpp1^WT^*, *Enpp1^asj^*, and *Enpp1^H362A^* mice; *n* = 4 *Enpp1^WT^*, 6 *Enpp1^asj^*, and 3 *Enpp1^H362A^* mice. (*I*) Plasma pyrophosphate concentrations in *Enpp1^WT^*, *Enpp1^asj^*, and *Enpp1^H362A^* mice; *n* = 6 *Enpp1^WT^*, 6 *Enpp1^asj^*, and 3 *Enpp1^H362A^* mice. (*J*) Ex vivo plasma ATP degradation at pH 7.5 assessed by luciferase assay after 45 min in *Enpp1^WT^*, *Enpp1^asj^*, and *Enpp1^H362A^* mice; *n* = 4 *Enpp1^WT^*, 4 *Enpp1^asj^*, and 5 *Enpp1^H362A^* mice. (*K* and *L*) In vitro ATP degradation comparing overexpressed ENPP1^WT^ and ENPP1^H362A^ as cell-surface proteins from cell lysate (*K*) and as secreted proteins from cell supernatant (*L*). For *C*–*J*, data are shown as the mean ± SD; *P* values were calculated by unpaired *t* test with Welch’s correction. *P < 0.05, **P < 0.01, ***P < 0.001, ****P < 0.0001.

We next created homozygous *Enpp1^H362A^* mice using CRISPR-based homologous recombination (*SI Appendix*, Fig. S5 *A* and *B*). After verifying the genotype of the mice (*SI Appendix*, Fig. S5*C*), we sought to confirm that these mice exhibited the expected enzymatic activity: intact ATP hydrolysis but defective cGAMP hydrolysis. We compared tissue lysates from *Enpp1^WT^* and *Enpp1^H362A^* mice to those from *Enpp1^asj^* mice, which harbor a point mutation that abolishes all ENPP1 activity (36). While tissue lysates from *Enpp1^WT^* mice rapidly degraded [^32^P]-cGAMP, tissue lysates from *Enpp1^H362A^* mice did not, mirroring the deficiency seen in *Enpp1^asj^* mice (Fig. 4*A*). In addition to the ENPP1 tethered to the surface of cells, ENPP1 is also secreted into the circulation, allowing cGAMP degradation to occur in the plasma (53, 54). Plasma from *Enpp1^WT^* mice readily degraded cGAMP, while plasma from *Enpp1^H362A^* mice did not (Fig. 4*B*). Despite the inability to degrade cGAMP, basal tissue cGAMP and *Ifnb1* transcript levels were generally low and not significantly different between *Enpp1^WT^*, *Enpp1^H362A^*, and *Enpp1^asj^* mice (*SI Appendix*, Fig. S5 *D* and *E*), suggesting that basal cGAMP signaling is not influenced by ENPP1. Finally, we assessed cGAMP degradation in vivo by subcutaneously injecting cGAMP into each mouse and measuring cGAMP concentration in the plasma by mass spectrometry. Thirty minutes after injection, cGAMP levels in the plasma of *Enpp1^H362A^* and *Enpp1^asj^* mice were >100-fold higher than in *Enpp1^WT^* mice (Fig. 4*C*), demonstrating that cGAMP hydrolysis is severely impaired in *Enpp1^H362A^* mice.

We then assessed ATP activity in these mice using liver lysates. The liver was the only organ suitable for these assays, as it expresses high levels of ENPP1, while ENPP1-independent ATP degradation predominates in other organs, precluding them from being used in this assay. Liver lysates from *Enpp1^asj^* mice had a marked deficiency in ATP degradation, but lysates from *Enpp1^H362A^* mice degraded ATP as well as those from *Enpp1^WT^* mice, confirming that tissue ENPP1^H362A^ retains the ability to degrade ATP (Fig. 4*D* and *SI Appendix*, Fig. S5*F*). Taking these data together, we find that *Enpp1^H362A^* mice are unable to degrade cGAMP but retain the ability to degrade ATP.

### *Enpp1^H362A^* Mice Do Not Exhibit the Severe Systemic Calcification Seen in ENPP1-Null Humans and Mice.

Inactivating mutations in ENPP1 cause progressive calcification of joints, vasculature, and soft tissue, leading to premature death in both mice (36) and humans; the human disease is known as generalized arterial calcification of infancy (37, 38, 55). It is hypothesized that this lethal aberrant calcification is due to the inability to degrade extracellular ATP, leading to a deficiency in PP_i_, which is known to regulate mineralization (55). However, since cGAMP was only recently identified as a substrate of ENPP1, it is unknown if impaired cGAMP metabolism also contributes to the systemic calcification observed in humans and mice.

As cGAMP degradation is uncoupled from extracellular ATP degradation in *Enpp1^H362A^* mice, they are an ideal model for determining the role of extracellular cGAMP in the progressive calcification seen in *Enpp1^asj^* mice. *Enpp1^H362A^* mice have a normal lifespan and readily breed, unlike the *Enpp1^asj^* mice, which have difficulty breeding beyond 2 to 3 mo of age due to worsening arthritis. Furthermore, *Enpp1^H362A^* mice do not exhibit the gross joint calcification or tissue calcification seen in *Enpp1^asj^* mice (Fig. 4 *E* and *F*). Taken together, these results suggest that extracellular cGAMP does not play a significant role in the phenotype seen in *Enpp1^asj^* mice and that this phenotype is likely due to the disruption of extracellular ATP metabolism.

*Enpp1^asj^* mice are reported to have normal plasma calcium and phosphate levels, while their plasma PP_i_ levels are significantly decreased due to their inability to degrade ATP (36). Like *Enpp1^WT^* and *Enpp1^asj^* mice, *Enpp1^H362A^* mice had normal plasma calcium and phosphate levels (Fig. 4 *G* and *H*). However, while *Enpp1^WT^* mice had a normal level of plasma PP_i_ (1.5 μM), *Enpp1^H362A^* and *Enpp1^asj^* mice had 300 nM and 60 nM plasma PP_i_, respectively (Fig. 4*I*). This was an unexpected finding, as *Enpp1^H362A^* liver lysate did not exhibit a defect in ATP degradation (Fig. 4*D*), raising the possibility that plasma ENPP1^H362A^ does not exhibit the same behavior as tissue ENPP1^H362A^. Indeed, *Enpp1^H362A^* mouse plasma was defective in ATP degradation, similar to *Enpp1^asj^* (Fig. 4*J* and *SI Appendix*, Fig. S5*G*). To test if this discrepancy was due to differences in plasma and tissue ENPP1^H362A^, we used our in vitro activity assay to compare secreted ENPP1 in cell supernatants with cell-surface ENPP1 present in cell lysates. Unlike the cell-surface ENPP1^H362A^, the secreted ENPP1^H362A^ was defective in ATP degradation (Fig. 4 *K* and *L* and *SI Appendix*, Fig. S5 *H* and *I*). Although we currently lack the ability to measure extracellular PP_i_ levels in tissues, we surmise that tissue PP_i_ is normal in *Enpp1^H362A^* mice and that tissue PP_i_, rather than plasma PP_i_, is important for preventing tissue calcification. Supporting this hypothesis, a recent study found that osteoblast-specific loss of ENPP1 led to increased bone volume, decreased trabecular spacing, and increased matrix calcification, without any change to plasma PP_i_ (56).

### Enhanced Extracellular cGAMP Signaling Confers Resistance to Herpes Simplex Virus-1 Infection.

Extracellular cGAMP has not been implicated in STING-related diseases outside of cancer. Since ENPP1 only regulates the extracellular cGAMP branch of STING signaling, it is unclear if the *Enpp1^H362A^* mice would be more resistant to viral infection. In contrast, there is a substantial body of evidence linking extracellular ATP signaling to viral infection (57, 58), including infection by the DNA virus herpes simplex virus (HSV)-1 (59). Therefore, we sought to investigate the relative contributions of extracellular ATP and cGAMP to the antiviral immune response by infecting *Enpp1^WT^*, *Enpp1^H362A^*, and *Enpp1^asj^* mice with a sublethal dose of HSV-1 using an established protocol (4).

**Fig. 5.**
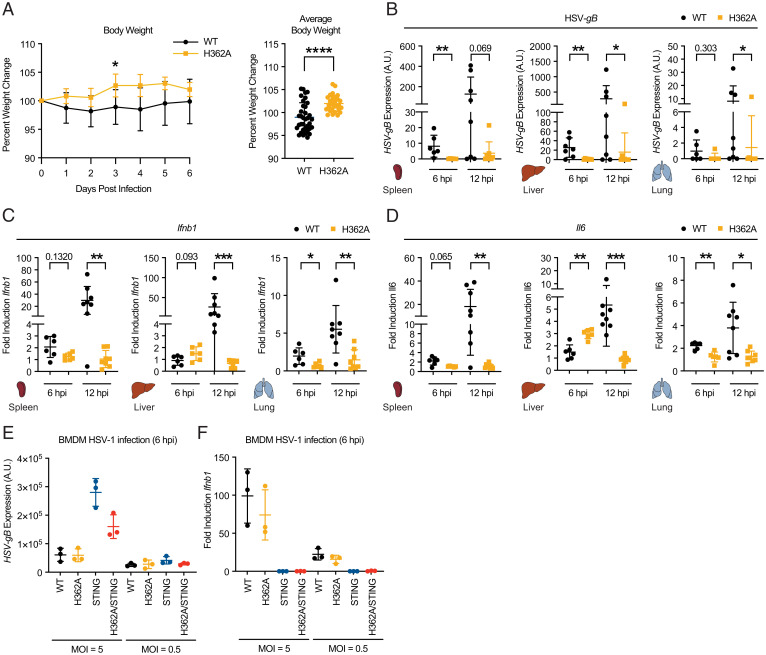
Enhanced extracellular cGAMP signaling confers resistance to HSV-1 infection. (*A*) Mice were infected with 2.5 × 10^7^ PFU per mouse HSV-1 through intravenous injection and body weight was monitored over 6 d. Percent body weight change is plotted for each day postinfection (*Left*) and as an average of days 1 to 6 postinfection (*Right*); *n* = 6 for *Enpp1^WT^* and *n* = 5 for *Enpp1^H362A^*. *B*–*D* Mice were infected with 2.5 × 10^7^ PFU per mouse of HSV-1 through intravenous injection and euthanized at 6 or 12 hpi. RNA was isolated from the spleen, liver, and lungs. qRT-PCR was performed to determine the expression levels of HSV-*gB* (*B*), *Ifnb1* (*C*), and *Il6* (*D*); *n* = 6 (6 hpi) or 8 (12 hpi) infected mice per genotype. (*E* and *F*) BMDMs isolated from *Enpp1^WT^*, *Enpp1^H362A^*, *Sting1*^−/−^, and *Sting1*^−/−^/*Enpp1^H362A^* mice were infected at a multiplicity of infection (MOI) = 0.5 or 5. qRT-PCR was performed to determine the expression levels of HSV-*gB* (*E*) and *Ifnb1* (*F*). For all qRT-PCR data, transcript levels were normalized to *Actb*. Cytokine transcript levels were also normalized to the average of two uninfected controls per genotype. Data are shown as the mean ± SD; *P* values were calculated using the nonparametric Mann–Whitney *U* test, appropriate for bimodal data. *P < 0.05, **P < 0.01, ***P < 0.001; ****P < 0.0001; *P* value is shown if between 0.05 and 0.1.

Compared to *Enpp1^WT^* mice, *Enpp1^H362A^* mice were more resistant to HSV-1 infection. *Enpp1^H362A^* mice did not experience the weight loss observed in *Enpp1^WT^* mice over the course of infection (Fig. 5*A*). *Enpp1^H362A^* mice also had lower levels of the viral transcript HSV-*gB* in the spleen, liver, and lung at 6 and 12 h postinfection (hpi) (Fig. 5*B*). This was accompanied by lower levels of replicating virus in *Enpp1^H362A^* spleen and kidney lysates (*SI Appendix*, Fig. S6*A*). In general, *Enpp1^H362A^* mice exhibited decreased expression of *Ifnb1* and the downstream cytokines *Il6* and *Cxcl10* in the liver, lung, and spleen, as well as decreased *Tnfa* in the liver (Fig. 5 *C* and *D* and *SI Appendix*, Fig. S6 *B* and *C*) at 6 and 12 hpi. As an exception, liver *Ifnb1* (Fig. 5*C*) and *Il6* (Fig. 5*D*) were higher in *Enpp1^H362A^* mice at 6 hpi. We hypothesize that the much higher levels of HSV in the liver compared to other organs (Fig. 5*B*) led to a slower decline in liver *Ifnb1* expression, allowing us to capture the time point when *Enpp1^H362A^* mice mounted a more robust immune response than *Enpp1^WT^* mice.

We hypothesize that the lower cytokine expression in *Enpp1^H362A^* mice at 12 hpi was due to faster viral clearance as a result of a more robust early immune response. It is also possible that as a plasma membrane protein, ENPP1^H362A^ reduced viral load by preventing or slowing viral entry. To distinguish between these two models, we first measured gene expression of mice at 6 d postinfection and observed no active infection in either genotype, suggesting that the infection was not simply delayed in *Enpp1^H362A^* mice (*SI Appendix*, Fig. S6 *D*–*H*). We then infected bone marrow-derived macrophages (BMDMs) isolated from *Enpp1^WT^* and *Enpp1^H362A^* mice to evaluate for any cell-intrinsic defects in viral entry or replication. *Enpp1^WT^* and *Enpp1^H362A^* BMDMs were both readily infected by HSV-1 (Fig. 5*E*) and produced *Ifnb1* (Fig. 5*F*). Resistance to infection and *Ifnb1* induction depended on STING in both genotypes (Fig. 5 *E* and *F*). These data indicate that the increased resistance to infection and decreased cytokine production observed in *Enpp1^H362A^* mice are not due to impaired viral entry or replication in *Enpp1^H362A^* cells.

Interestingly, *Enpp1^asj^* mice did not significantly differ from *Enpp1^WT^* mice in their response to HSV-1 infection (*SI Appendix*, Fig. S7 *A*–*F*), except for a decrease in the expression of *Ifnb1* in the lung (*SI Appendix*, Fig. S7*B*) and *Il6* in the liver (*SI Appendix*, Fig. S7*C*). It is possible that impaired ATP metabolism or the overall poor health of *Enpp1*^asj^ mice compromises the antiviral effect of extracellular cGAMP-STING signaling.

Because *Enpp1^H362A^* mice are deficient in plasma ATP hydrolysis, we measured plasma ATP before and after infection to rule out the possibility that increased plasma ATP contributes to controlling viral infection by activating purinergic signaling. We observed generally low (below the *K*_d_ toward purinergic receptors) and not significantly different levels of plasma ATP in *Enpp1^H362A^* and *Enpp1^WT^* mice (*SI Appendix*, Fig. S7*G*). These data demonstrate that enhanced extracellular cGAMP alone is protective against HSV-1 infection, and that its hydrolysis by ENPP1 normally dampens this protective signal. Interestingly, we did not detect cGAMP in the plasma of mice infected with HSV-1, with an assay detection limit of 85 pg/mL (125 pM) (*SI Appendix*, Fig. S7*H*). The lack of circulating cGAMP suggests that extracellular cGAMP is acting locally as a paracrine immunotransmitter to confer resistance to HSV-1.

### Enhanced Extracellular cGAMP Signaling Exacerbates Radiation-Induced Inflammation.

**Fig. 6.**
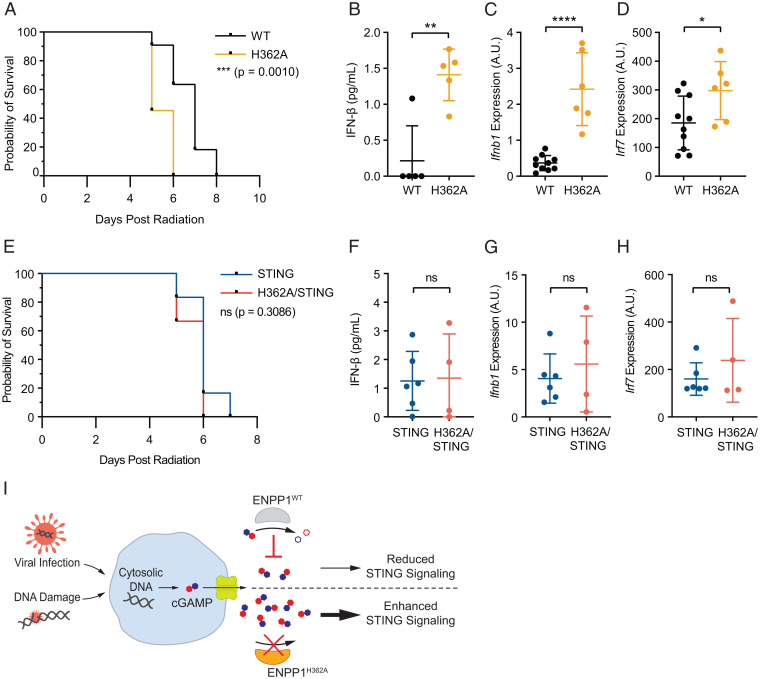
Enhanced extracellular cGAMP exacerbates radiation-induced inflammation. (*A*–*D*) *Enpp1^WT^* and *Enpp1^H362A^* mice were irradiated with 8 or 9 Gy of total body irradiation and then weighed daily. Mice were euthanized if they exhibited greater than 20% weight loss for 2 consecutive days; *n* = 11 mice per genotype (6 mice at 8 Gy and 5 mice at 9 Gy). (*A*) Kaplan–Meier plot showing the probability of survival for each genotype. *P* value was calculated using a log rank (Mantel–Cox) test. (*B*) Blood was drawn from each mouse 5 d postirradiation. Plasma IFN-β concentration was determined using a high-sensitivity IFN-β ELISA. *P* value was calculated using an unpaired *t* test. (*C* and *D*) Spleens were harvested from euthanized mice at experiment endpoint. RNA was extracted and qRT-PCR was used to determine expression levels of *Ifnb1* (*C*) and *Irf7* (*D*). One splenic H362A sample was excluded from *C* and *D* as an outlier based on the ROUT method (Q = 1%). (*E*–*H*) *Sting1*^−/−^ and *Sting1*^−/−^/*Enpp1^H362A^* mice were irradiated with 9 Gy of total body irradiation and then weighed daily; *n* = 6 mice per genotype. Procedures described in *A*–*D* were repeated to obtain plasma IFN-β concentrations (*F*) and splenic expression levels of *Ifnb1* (*G*) and *Irf7* (*H*). *P* values were calculated using an unpaired *t* test. *P < 0.05, **P < 0.01, ****P < 0.0001. (*I*) Model of cGAMP as a paracrine immunotransmitter regulated by ENPP1 during viral infection and radiation-induced inflammation. Infected or irradiated cells produce and export cGAMP in response to cytosolic double-stranded DNA. When ENPP1^WT^ is present, extracellular cGAMP is degraded, diminishing STING pathway activation. When cGAMP-specific ENPP1^H362A^ is present, extracellular cGAMP accumulates, leading to an enhanced response to viral infection or an exacerbated response to radiation-induced inflammation.

As extracellular cGAMP enhances both the antiviral and anticancer immune responses (22) and ENPP1 negatively regulates extracellular cGAMP, it begs the question: why is the ability of ENPP1 to hydrolyze cGAMP conserved throughout evolution? We hypothesized that paracrine extracellular cGAMP is a pivotal STING activator in extensive or sustained DNA damage and that ENPP1 plays an indispensable role in preventing the subsequent hyperactive and potentially damaging immune response. To test this hypothesis, we used total-body ionizing radiation exposure in our *Enpp1^H362A^* mice and *Enpp1^WT^* mice to generate systemic DNA damage and inflammation (5, 6, 9, 22). After 8 to 9 Gy of total body irradiation, the *Enpp1^WT^* and *Enpp1^H362A^* mice were weighed daily and euthanized when they reached the humane endpoint of greater than 20% weight loss for 2 consecutive days. Strikingly, the *Enpp1^H362A^* mice exhibited significantly shortened survival compared to *Enpp1^WT^* mice (Fig. 6*A* and *SI Appendix*, Fig. S8). This was accompanied by induction of cytokines and transcription factors associated with STING activation, including increased plasma IFN-β (Fig. 6*B*), increased splenic expression of *Ifnb1* (Fig. 6*C*), and increased splenic expression of the downstream transcription factor *Irf7* (Fig. 6*D*). To confirm that the enhanced radiation toxicity in *Enpp1^H362A^* mice is STING-dependent, we compared the response to radiation in *Sting1*^−/−^/*Enpp1^H362A^* and *Sting1*^−/−^ mice. Without an intact STING pathway, these mice showed no difference in survival or cytokine production in response to radiation (Fig. 6 *E*–*H*), suggesting that the enhanced radiation toxicity in these mice is STING-dependent.

## Discussion

In this study we identified and characterized ENPP1^H362A^, a single histidine mutation in ENPP1 that shows remarkable substrate selectivity: it abolishes 2′3′-cGAMP degradation but leaves activity toward ENPP1’s other substrates intact. Remarkably, this histidine is nearly 100% conserved throughout evolutionary history down to bacteria. Previously, the only link between cGAS-STING and NPP was in higher mammals, where ENPP1 degrades extracellular cGAMP to dampen STING activation. We found that *Xac* NPP not only degrades cyclic dinucleotides but also prefers 2′-5′–linked over 3′-5′–linked cyclic dinucleotides. Furthermore, the conserved histidine is required for degrading the 2′-5′ but not 3′-5′ phosphodiester linkage. Although 3′-5′–linked bacterial cyclic dinucleotides are ubiquitous signaling molecules that are important for cell cycle regulation, biofilm formation, motility, and virulence (60, 61), 2’-5′–linked cyclic dinucleotides were thought to be unique to metazoans until the recent discoveries of bacterial 2′-5′–linked cyclic oligonucleotides (47) and 3′2′-cGAMP (48). We predict that 2′-5′–linked cyclic dinucleotides and oligonucleotides are a general bacterial antiviral defense mechanism and that NPP is a negative regulator of these systems. We propose that mutations of the conserved histidine can be introduced into a wide variety of eukaryotic and prokaryotic organisms to study the pathophysiology of extracellular second messengers with 2′-5′ linkages.

Although previous work has linked cGAMP-STING signaling to defense against HSV-1 infection (62), the role of extracellular cGAMP in anti–HSV-1 defense has only been tested using mice harboring a genetic deletion of the LRRC8 channels (33). The LRRC8 channels transport chloride, aspartate, glutamate, and other organic osmolytes in addition to cGAMP (31, 33). As disrupting the transport of chloride and amino acids can significantly impact the immune response to viral infection, we used the *Enpp1^H362A^* mouse strain to specifically investigate the role of extracellular cGAMP in antiviral defense. We found that *Enpp1^H362A^* mice are significantly more resistant to HSV-1 infection, definitively demonstrating that extracellular cGAMP plays a key role in viral defense.

Given its negative roles in the antiviral and anticancer immune responses, it is puzzling that the cGAMP hydrolysis activity of the NPP family is so well-conserved. Our discovery of ENPP1’s role in controlling the damage caused by systemic STING overactivation sheds light on this question. The loss of ENPP1’s regulation of extracellular cGAMP significantly increased cytokine production in response to ionizing radiation, resulting in drastically decreased overall survival. These results suggest that extracellular cGAMP exacerbates STING-mediated radiation toxicity and that multicellular organisms rely on ENPP1 to control systemic inflammation. We hypothesize that extracellular cGAMP also contributes to the pathology of many other STING-mediated inflammatory conditions, including autoimmunity (13–16), neurodegeneration (10, 11), myocardial infarction (12), and acute pancreatitis (63). Therefore, ENPP1 may be a vital anti-inflammatory innate immune checkpoint, explaining its remarkable evolutionary conservation (Fig. 6*I*). Future studies using our *Enpp1^H362A^* mice are warranted to assess the involvement of extracellular cGAMP in these conditions and to evaluate the role of ENPP1 as an innate immune checkpoint. Additionally, the regulation of ENPP1 expression and activity in chronic diseases remains an open question.

As we expand our knowledge of the importance of extracellular cGAMP signaling to new diseases and physiological settings, the ability to regulate extracellular cGAMP will provide new therapeutic opportunities to manipulate and treat these diseases. ENPP1 inhibitors, and perhaps even cGAMP-specific ENPP1 inhibitors, may be therapeutically beneficial during viral infection, while administering recombinant ENPP1 or blocking cGAMP import may reduce extracellular cGAMP-associated inflammation.

## Methods

See *SI Appendix* for detailed methods.

### Cell Culture.

HEK 293T *ENPP1^−/−^* cells were generated in a previous study (22). Vero and Expi293F cell lines were gifts from Peter Kim, Stanford University, Stanford, CA. The 293T *ENPP1^−/−^* and Vero cell lines were maintained in a 5% CO_2_ incubator at 37 °C in DMEM (Corning Cellgro) supplemented with 10% FBS (Atlanta Biologics) and 100 U mL^−1^ penicillin-streptomycin (Thermo Fisher). The Expi293F cell line was maintained in an 8% CO_2_ incubator at 37 °C shaking in baffled flasks, in a mixture of 66% FreeStyle Expression Media (Thermo Fisher) and 33% Expi293 Expression Media (Thermo Fisher). All cell lines tested negative for mycoplasma contamination.

### Recombinant DNA.

The pcDNA3-mouseENPP1-FLAG plasmid was synthesized by Genscript. The *Xac* NPP-MBP-pMAL plasmid was a gift from Daniel Herschlag, Stanford University, Stanford, CA (46). The single-point mutations were introduced using QuikChange mutagenesis (Agilent) and verified by sequencing the region of the mutation. Primers used for mutagenesis are shown in *SI Appendix*, Table S3.

### Enzyme Activity Assays.

All enzyme activity assays were performed with protein source indicated, including cell lysate, recombinant protein, organ lysate, or plasma.

### Recombinant Mouse ENPP1 and *Xac* NPP Purification.

His-tagged mouse ENPP1 was expressed in Expi293F cells and purified by cobalt-based immobilized metal ion affinity chromatography (IMAC). His-tagged Xac NPP was expressed in *Escherichia coli* BL21(DE3) cells and purified by cobalt-based IMAC and anion-exchange chromatography.

### NPP Family Sequence Alignment and Signal Peptide Prediction.

Representative species were selected from classic model organisms and the EMBL-EBI protein family (Phosphodiest PF01663). Multiple sequence alignment was performed using MAFFT and visualized using Jalview. Protein accession numbers are listed in *SI Appendix*, Table S4. To determine the histidine conservation in all known NPP sequences, 998 eukaryotic, 1,000 bacterial, and 584 archaeal NPP protein sequences were downloaded from Uniprot and pairwise-aligned using MUSCLE alignment (64). The histidine corresponding to H362 in mouse ENPP1 was identified and the percent conservation was determined for Eukaryota, Bacteria, and Archaea. Signal peptides were predicted using SignalP-5.0 and transmembrane domains were predicted using TMHMM (DTU Bioinformatics).

### Crystallization of *Xac* NPP^T90A^ with pApG and *Xac* NPP^H214A^ Apo.

*Xac* NPP^T90A^ and *Xac* NPP^H214A^ were expressed, purified, and crystallized as described previously (42).

### Mouse Models.

C57BL/6J (Stock #000664), C57BL/6J-*Enpp1^as^*^j^/GrsrJ (Stock #012810), and C57BL/6J-*Sting1^gt^*/J (Stock #017537) mice were purchased from the Jackson Laboratory. *Enpp1*^H362A^ were generated and characterized in house and bred with C57BL/6J-*Sting1^gt^*/J mice to generate *Sting1*^−/−^*Enpp1^H362A^* mice. Male and female mice were included in every experiment, unless otherwise noted. Mice were maintained at Stanford University in compliance with the Stanford University Institutional Animal Care and Use Committee regulations. All procedures were approved by the Stanford University Administrative Panel on Laboratory Animal Care.

### In Vivo and In Vitro HSV-1 Infection Models.

For in vivo and in vitro HSV-1 infection models, 2.5 × 10^7^ PFU of HSV-1 was diluted in 100 μL PBS and injected intravenously into the tail vein of 6- to 9-wk-old mice. After 6 h, 12 h, or 6 d, the mice were euthanized in a CO_2_ chamber and blood and organs were harvested.

### Total Body Irradiation Mouse Model.

Male and female 8- to 12-wk-old mice were irradiated with either 8 or 9 Gy using a 225-kVp cabinet X-ray irradiator with a 0.5-mm Cu filter (IC-250, Kimtron Inc.). The mice were weighed daily and were euthanized if they met the humane endpoint of greater than 20% weight loss for 2 consecutive days.

### Statistical Analysis.

All statistical tests were performed using GraphPad Prism software and are noted in the figure legends. Data are presented as the mean ± SD, unless otherwise stated.

## Supplementary Material

Supplementary File

## Data Availability

All study data are included in the article and *SI Appendix*.
