# Supplementary material for: ENPP1’s regulation of extracellular cGAMP is a ubiquitous mechanism of attenuating STING signaling
Source: Proc Natl Acad Sci U S A. 2022 May 19;119(21):e2119189119. doi: 10.1073/pnas.2119189119 (PMC9173814; doi:10.1073/pnas.2119189119)
Supplement: Supplementary File [file pnas.2119189119.sapp.pdf]

Supplementary Information:

ENPP1's regulation of extracellular cGAMP is a ubiquitous mechanism of attenuating STING signaling

Jacqueline A. Carozza[1,2,7], Anthony F. Cordova[1,3,7], Jenifer Brown[1,4], Yasmeen AlSaif[1,5], Volker Böhnert[1,6], Gemini Skariah[1,6], Daniel Fernandez[1,6], Xujun Cao[1,2], Rachel E. Mardjuki[1,2], Lingyin Li[1,6]*

[1]ChEM-H Institute, [2]Department of Chemistry, [3]Cancer Biology Program, [4]Department of Biophysics, [5]Department of Biology, [6]Department of Biochemistry, Stanford University, Stanford, CA 94301

[7]These authors contributed equally

*Correspondence: lingyinl@stanford.edu

Table of Contents:

Supplementary Fig. 1 Kinetic analysis and expression of ENPP1 guanosine-binding pocket mutations.
Supplementary Fig. 2 Kinetic analysis and expression of ENPP1 zinc binder mutations.
Supplementary Fig. 3 Kinetic analysis and expression of ENPP1 H362X mutations.
Supplementary Fig. 4 Bacterial NPP selectively cleaves 2'-5' linkages in cyclic dinucleotides using a conserved histidine.
Supplementary Fig. 5 $Enpp1^{H362A}$ mice do not exhibit the severe systemic calcification seen in ENPP1-null humans and mice.
Supplementary Fig. 6 Enhanced extracellular cGAMP signaling confers resistance to HSV-1 infection.
Supplementary Fig. 7 HSV-1 infection of $Enpp1^{asj}$ and $Enpp1^{H362A}$ mice.
Supplementary Fig. 8 Enhanced extracellular cGAMP exacerbates radiation-induced inflammation.

Supplementary Appendix 1. Detailed Methods

1. Synthesis and purification of cGAMP and [^{32}P]-cGAMP
2. Enzyme assays
   a) cGAMP
   b) ATP
   c) GTP, CTP, and UTP
   d) Additional cyclic dinucleotides
3. Recombinant mouse ENPP1 purification
4. Preparing ENPP1-transfected cell lysates
5. Recombinant *Xac* NPP purification
6. Crystallization of *Xac* NPP^{T90A} with pApG and *Xac* NPP^{H214A} apo
7. Computational modeling of substrates
8. Generation and characterization of the transgenic $Enpp1^{H362A}$ mouse strain
   a) GAMP ELISA for basal cGAMP detection.
   b) Histology sectioning and staining.
   c) Plasma chemistry.
   d) *In vivo* cGAMP metabolism.
9. HSV-1 purification
10. *In vivo* and *in vitro* HSV-1 infection models
11. HSV-1 plaque assays
12. Mouse BMDM isolation and infection
13. RT-qPCR
14. cGAMP ELISA for plasma cGAMP measurement
15. Total body irradiation mouse model

Supplementary Table 1. Crystallographic data collection and refinement statistics
Supplementary Table 2. Intermolecular contacts between pApG/AMP and *Xac* NPP^{T90A}
Supplementary Table 3. Oligonucleotides used in this study
Supplementary Table 4. Protein Accession Numbers for Representative Species

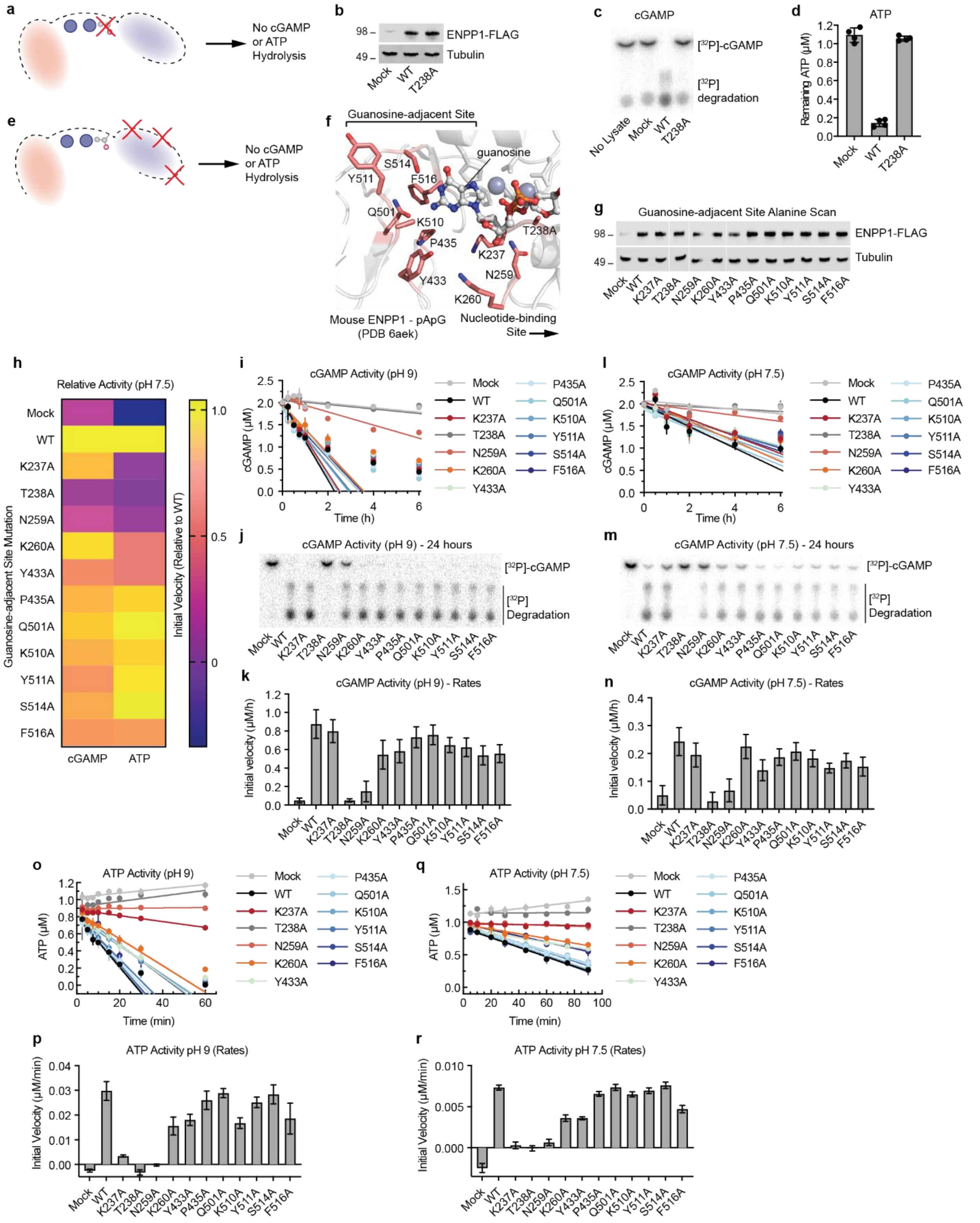

Supplementary Fig. 1 Kinetic analysis and expression of ENPP1 guanosine-binding pocket mutations. a Schematic illustration of mutations (red X's) of the nucleophile T238 (ball and stick), leading to no substrate hydrolysis. **b** Expression of $ENPP1^{WT}$ and $ENPP1^{T238A}$ mutation, representative of 3 independent experiments. **c** TLC cGAMP degradation assay for

ENPP1 mutations expressed in cell lysate. Representative data shown from 3 independent reactions. **d** Luciferase-based ATP degradation assay for ENPP1 mutations expressed in cell lysate. *n* = 3 independent reactions, mean ± SD. **e** Schematic illustration of mutations (red X's) of the nucleotide-binding site (blue), leading to no substrate hydrolysis. **f** Mouse ENPP1 (gray cartoon) in complex with pApG (gray ball and sticks) (PDB:6aek). Guanosine-adjacent residues are colored salmon. **g** Expression of ENPP1 guanosine-binding pocket mutations, representative of 2 independent experiments. **h** Heat map showing the initial velocity relative to WT of guanosine-binding pocket mutations for the substrates cGAMP and ATP at pH 7.5. **i-n** cGAMP degradation kinetics of ENPP1 guanosine-binding pocket mutations at pH 9 (**i-k**) and pH 7.5 (**l-n**). Cell lines were transfected with the indicated ENPP1 mutations. Lines in (**i**) and (**l**) represent linear fits of kinetic data during the linear portion of the reaction. Time = 0 to 1 h for (**i**) and time = 0 to 6 h for (**l**). Data is representative of 2 independent experiments. These linear fits were plotted in bar graphs in (**k**) and (**n**) showing the best fit slope ± SD of the fit and were used to make heat maps shown in Fig. 1d and (**h**). TLCs in (**j**) and (**m**) depict reaction progress after 24 hours. **o-r** ATP degradation kinetics of ENPP1 guanosine-binding pocket mutations at pH 9 (**o-p**) and pH 7.5 (**q-r**). Cell lines were transfected with the indicated ENPP1 mutations. Lines in (**o**) and (**q**) represent linear fits of kinetic data during the linear portion of the reaction. Time = 0 to 20 min for (**o**) and time = 0 to 90 min for (**q**). *n* = 4 independent reactions, mean ± SD shown with some error bars too small to visualize. These linear fits were plotted in bar graphs in (**p**) and (**r**) showing the best fit slope ± SD of the fit and were used to make heat maps shown in Fig. 1d and (**h**).

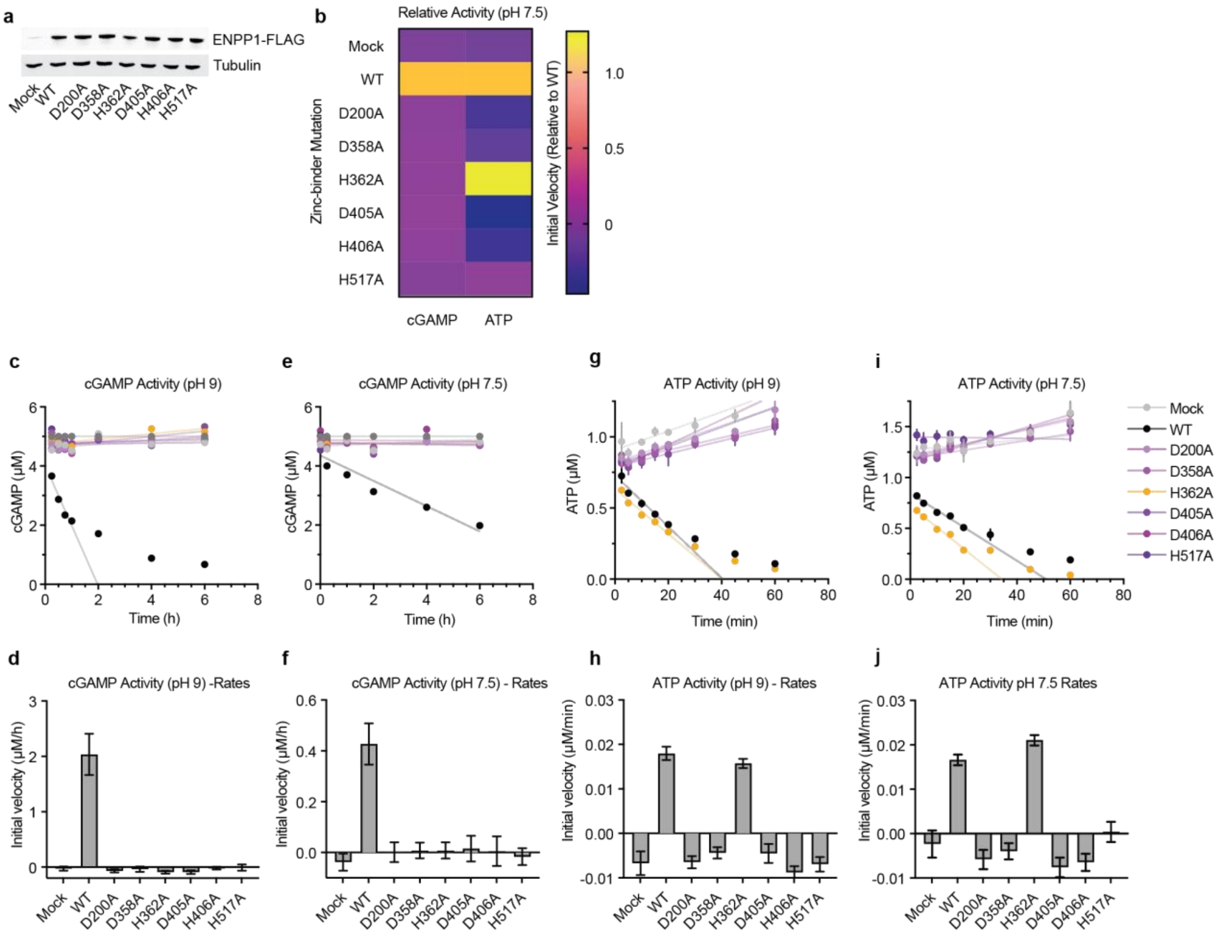

Supplementary Fig. 2 Kinetic analysis and expression of ENPP1 zinc binder mutations. a Expression of ENPP1 zinc-binding residue mutations, representative of 2 independent experiments. **b** Heat map showing the initial velocity relative to WT of zinc-binding residue mutations for the substrates cGAMP and ATP at pH 7.5. The mean initial velocity was calculated from a linear fit of the degradation reactions during early time points. *n* = 3 independent reactions. **c-f** cGAMP degradation kinetics of ENPP1 zinc-binding residue mutations at pH 9 (**c–d**) and pH 7.5 (**e–f**). Cell lines were transfected with the indicated ENPP1 mutations. Lines in (**c**) and (**e**) represent linear fits of kinetic data during the linear portion of the reaction. Time = 0 to 45 min for (**c**) and time = 0 to 6 h for (**e**). Data is representative of 2 independent experiments. These linear fits were plotted in bar graphs in (**d**) and (**f**) showing the best fit slope ± SD of the fit and were used to make heat maps shown in Fig. 1g and (**b**). **g-j** ATP degradation kinetics of ENPP1 zinc-binding residue mutations at pH 9 (**g-h**) and pH 7.5 (**i-j**). Cell lines were transfected with the indicated ENPP1 mutations. Lines in (**g**) and (**i**) represent linear fits of kinetic data during the linear portion of the reaction (time = 0 to 20 min). *n* = 3 independent reactions, mean ± SD with some error bars too small to visualize. These linear fits were plotted in bar graphs in (**h**) and (**j**) showing the best fit slope ± SD of the fit and were used to make heat maps shown in Fig. 1g and (**b**).

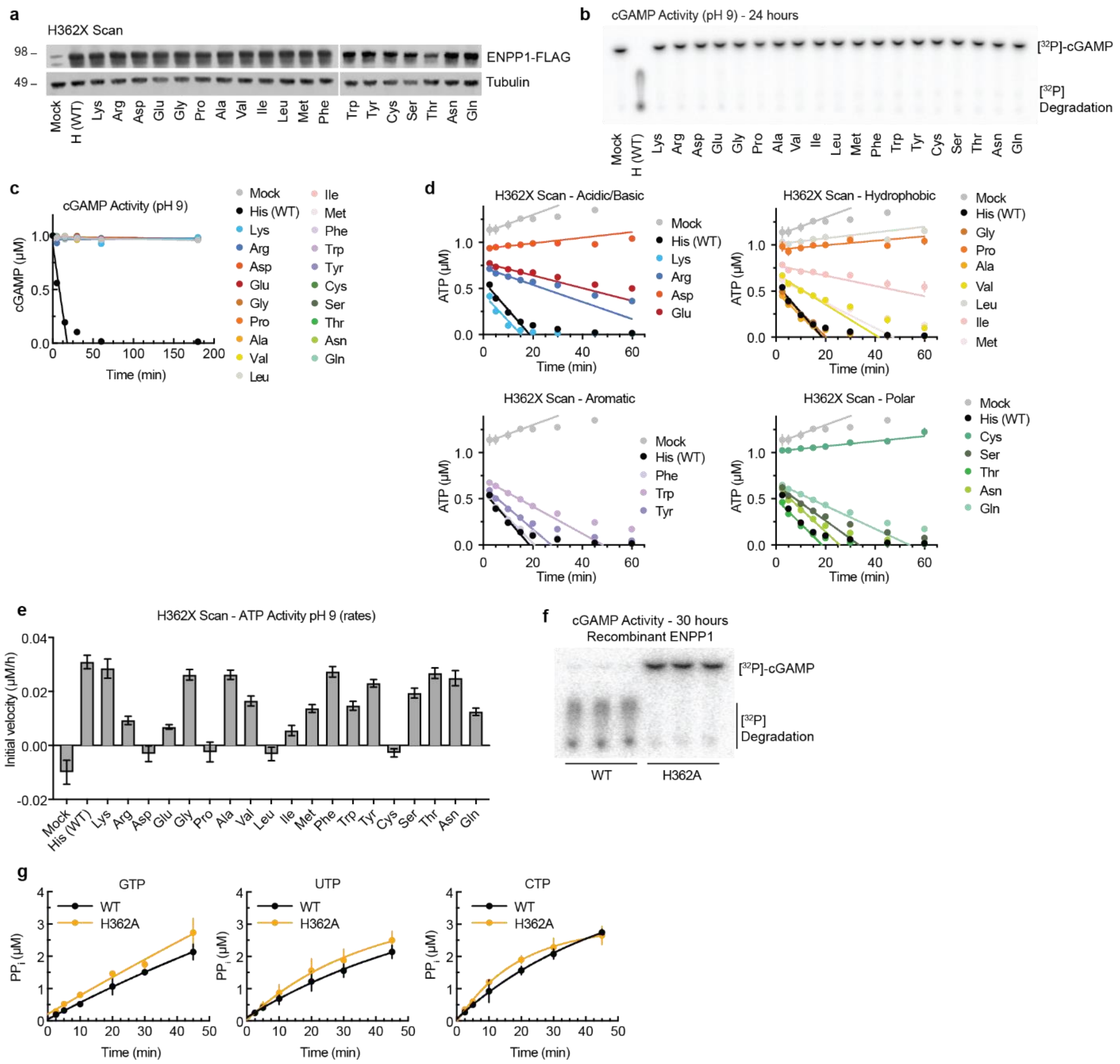

Supplementary Fig. 3 Kinetic analysis and expression of ENPP1 H362X mutations. a Expression of ENPP1 H362X mutations (where X represents the indicated amino acid), representative of 2 independent experiments. **b-c** cGAMP degradation kinetics of ENPP1 H362X mutations at pH 9. Cell lines were transfected with the indicated ENPP1 mutations. **b** TLC showing cGAMP degradation after 24 hours at pH 9. **c** Linear fits of kinetic data during the linear portion of the reaction (time = 0 to 30 minutes). **d** ATP degradation kinetics of ENPP1 H362X mutations at pH 9, organized by amino acid class. Cell lines were transfected with the indicated ENPP1 mutations. Lines represent linear fits of kinetic data during the linear portion of the reaction (time = 0 to 15 min). *n* = 3 independent reactions, mean ± SD with some error bars too small to visualize. **e** ATP activity rates for ENPP1 H362X mutations plotted from linear fits shown in (**d**). *n* = 3 independent reactions, best fit slope ± SD of the fit from (**d**). **f** TLC of cGAMP degradation comparing recombinant $ENPP1^{WT}$ to $ENPP1^{H362A}$. *n* = 3 independent reactions. **g** Kinetic analysis of GTP, UTP, and CTP monitoring pyrophosphate production using recombinant purified ENPP1. *n* = 2 independent reactions, mean ± SD.

a

Kingdom	Histidine Conservation
Eukaryota	100% (998/998)
Bacteria	99.9% (994/1000)
Archaea	53.9% (315/584)

b

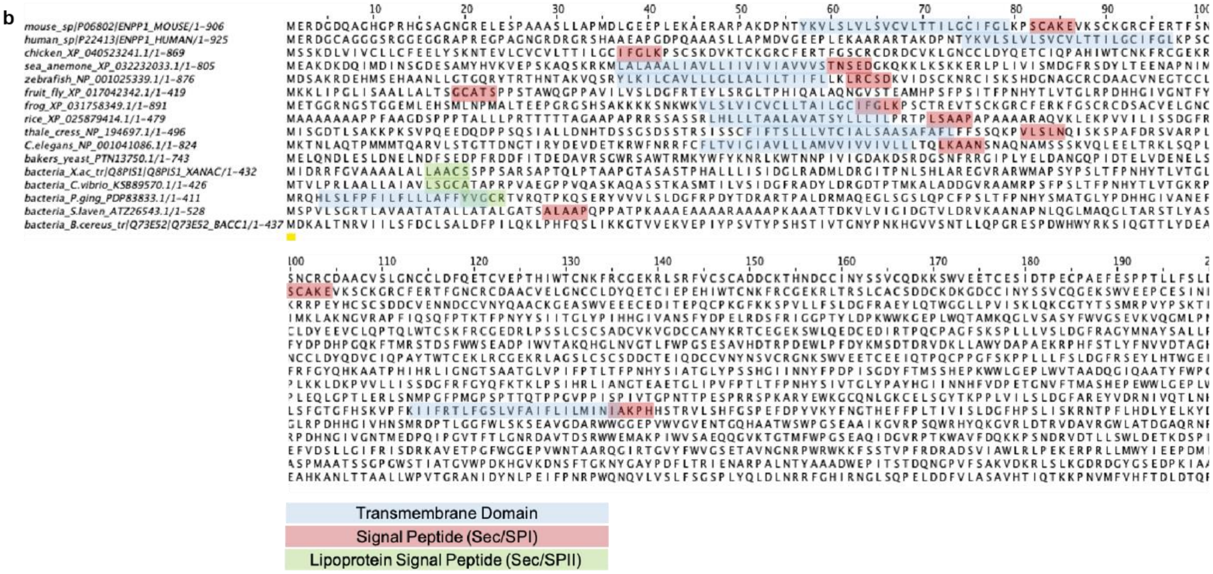

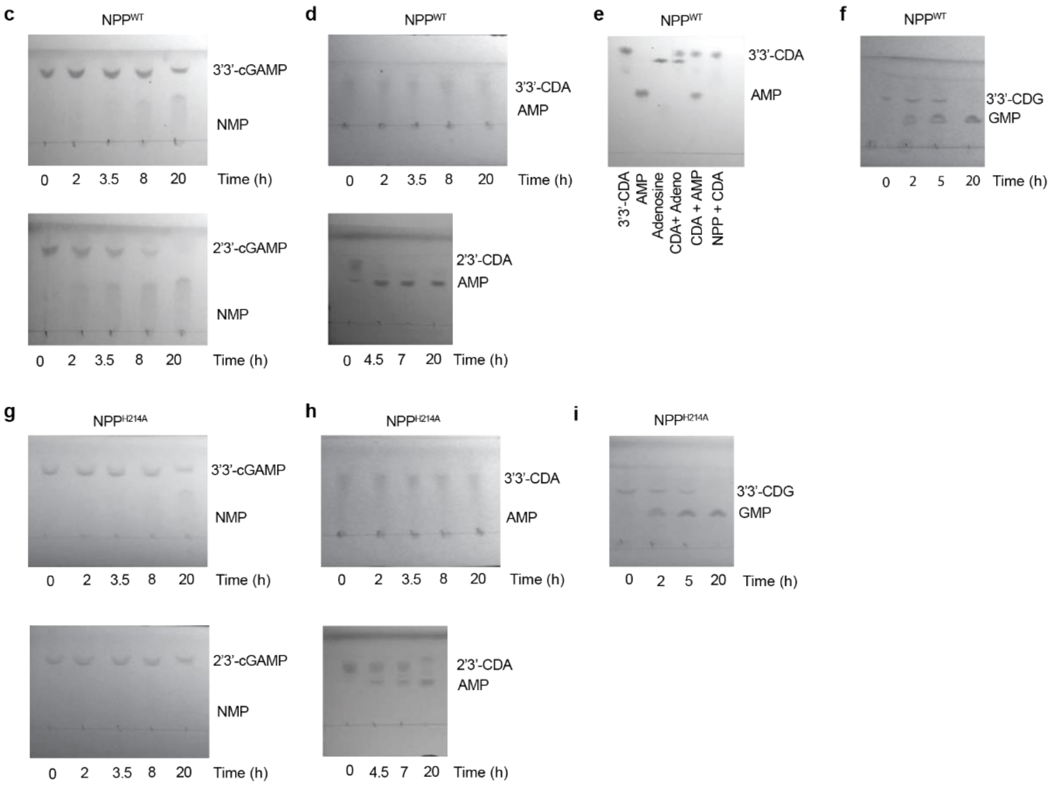

Supplementary Fig. 4 Bacterial NPP selectively cleaves 2’-5’ linkages in cyclic dinucleotides using the conserved histidine. a 998 eukaryotic, 1000 bacterial, and 584 archaeal NPP protein sequences were downloaded from Uniprot and pairwise aligned using MUSCLE alignment. The histidine corresponding to H362 in mouse ENPP1 was identified and

the percent conservation was determined for Eukaryota, Bacteria, and Archaea. **b** Signal peptides (red boxes) or lipoprotein signal peptides (blue boxes) for selected species predicted by SignalP5.0. No signal peptide was found for *B. cereus*. **c** TLCs showing the degradation of 3'3'-cGAMP (top) and 2'3'-cGAMP (bottom) by 1.5 µM *Xac* NPP^{WT} over the indicated times. **d** TLCs showing the degradation of 3'3'-CDA (top) and 2'3'-CDA (bottom) by 1.5 µM (3'3'-CDA) or 0.5 µM (2'3'-CDA) *Xac* NPP^{WT} over the indicated times. **e** TLC showing degradation of 3'3'-CDA by 1.5 µM *Xac* NPP^{WT} after 72 h. No degradation was observed. **f** TLC showing the degradation of 3'3'-CDG by 1.5 µM *Xac* NPP^{WT} over the indicated times. It was not possible to resolve 2'3'-CDG degradation using TLC under a variety of running conditions. **g** TLCs showing the degradation of 3'3'-cGAMP (top) and 2'3'-cGAMP (bottom) by 1.5 µM *Xac* NPP^{H214A} over the indicated times. **h** TLCs showing the degradation of 3'3'-CDA (top) and 2'3'-CDA (bottom) by 1.5 µM (3'3'-CDA) or 0.5 µM (2'3'-CDA) *Xac* NPP^{H214A} over the indicated times. **i** TLC showing the degradation of 3'3'-CDG by 1.5 µM *Xac* NPP^{H214A} over the indicated times. It was not possible to resolve 2'3'-CDG degradation using TLC under a variety of running conditions.

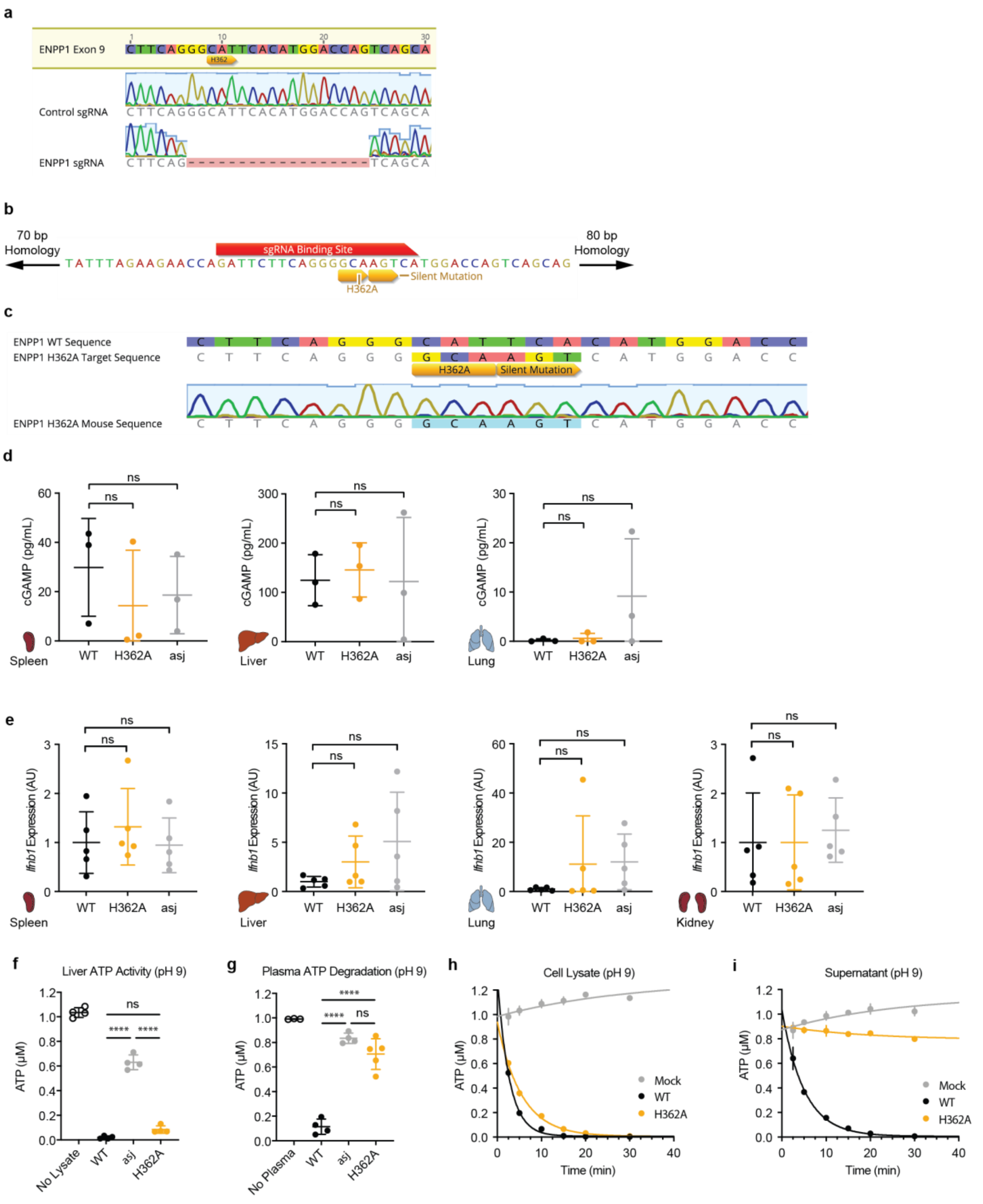

Supplementary Fig. 5. *Enpp1^{H362A}* mice do not exhibit the severe systemic calcification seen in ENPP1-null humans and mice. a A single-guide RNA (sgRNA) was designed to target the region near H362A in exon 9 of Enpp1. The sgRNA and Cas9 were introduced into 4T1 cells through lentiviral transduction. The cells were then sequenced to determine editing efficiency at the intended cleavage site. There was no evidence of editing in cells transduced with a control sgRNA, while there was a large deletion in cells transduced with the *Enpp1*

sgRNA. **b** Diagram indicating the sequence of the donor ssDNA used to generate the H362A point mutation through homologous recombination. Silent mutations were introduced downstream of H362 to prevent Cas9 from recognizing the cleavage site following successful recombination. **c** Genomic DNA sequencing from one of the *Enpp1*^{H362A} mice. The sequencing indicated that this mouse harbored a homozygous H362A point mutation in *Enpp1*, as well as the point mutations indicated in (**b**). **d** A cGAMP ELISA was performed to measure basal cGAMP in the spleen, kidney, liver, and lung. There was no detectable cGAMP in any of the kidneys, so they were omitted from analysis. $n = 3$ mice per genotype. *p* values were calculated by unpaired *t* test; $^{*}p < 0.05$. **e** RT-qPCR was performed to measure basal *Ifnb1* in the spleen, kidney, liver, and lung. $n = 5$ mice per genotype. *p* values were calculated by unpaired *t* test. **f** *Ex vivo* liver lysate (1 mg/mL) ATP degradation at pH 9 assessed by luciferase assay after 20 minutes in *Enpp1*^{H362A}, *Enpp1*^{asj}, and *Enpp1*^{H362A} mice. $n = 4$ *Enpp1*^{H362A}, 4 *Enpp1*^{asj}, and 4 *Enpp1*^{H362A} mice. **g** *Ex vivo* plasma ATP degradation at pH 9 assessed by luciferase assay after 45 minutes in *Enpp1*^{WT}, *Enpp1*^{asj}, and *Enpp1*^{H362A} mice. $n = 4$ *Enpp1*^{WT}, 4 *Enpp1*^{asj}, and 5 *Enpp1*^{H362A} mice. Data are shown as the mean ± SD. *p* values were calculated by unpaired *t* test with Welch's correction. $^{****}p < 0.0001$. **h-i** *In vitro* ATP degradation comparing overexpressed ENPP1^{WT} and ENPP1^{H362A} as cell-surface proteins from cell lysate (**h**) and as secreted protein from cell supernatant (**i**) at pH 9.

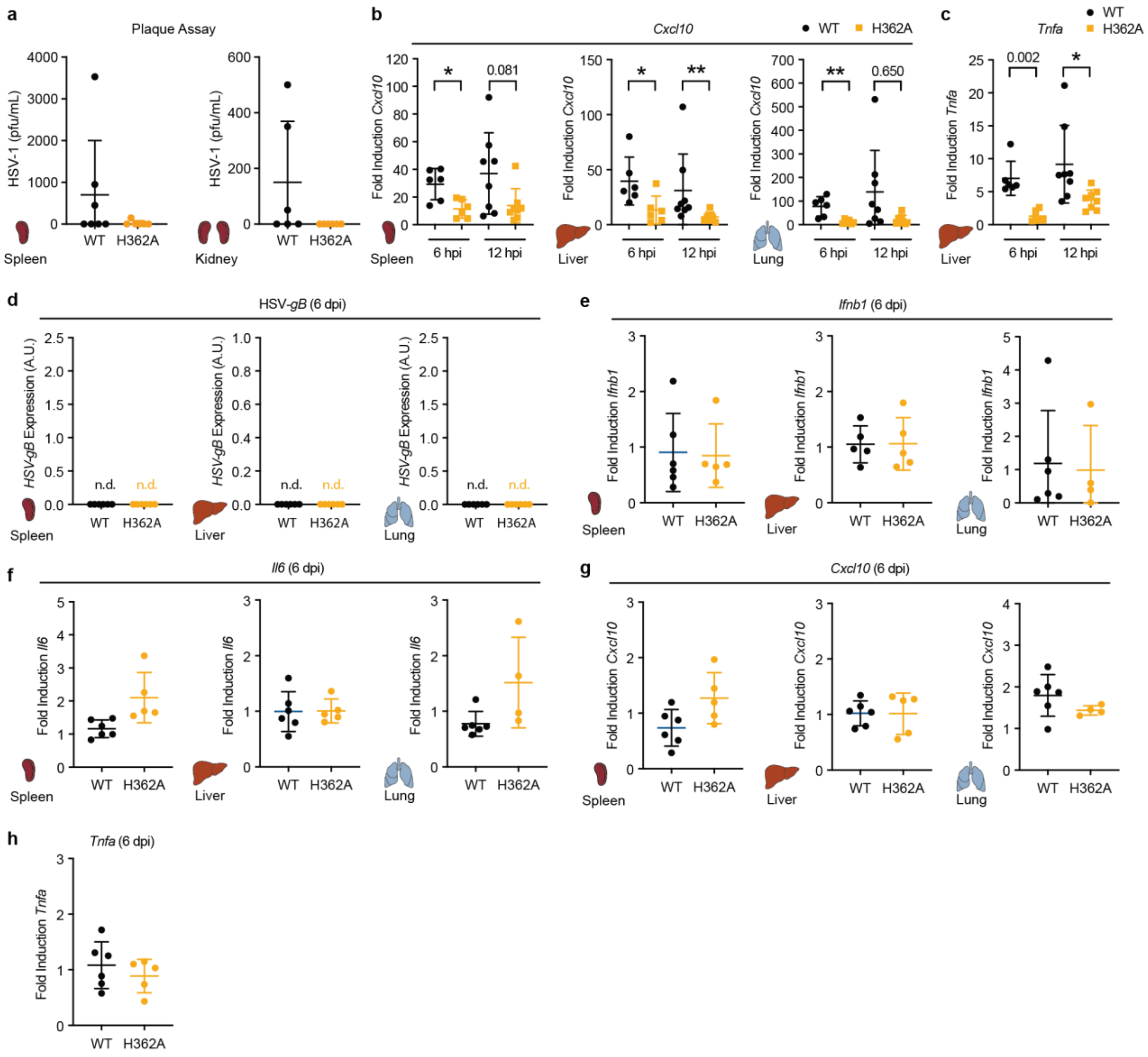

Supplementary Fig. 6 Enhanced extracellular cGAMP signaling confers resistance to HSV-1. a-c Mice were inoculated with 2.5 x 10^7 PFU/mouse HSV-1 through intravenous injection. The mice were euthanized at 6 or 12 hpi and organs were isolated for plaque assays or total RNA isolation. *n* = 6 (6 hpi) or 8 (12 hpi) infected mice per genotype (as previously described in Fig. 5). **a** Plaque assays of spleen and kidney lysates from *Enpp1^{WT}* and *Enpp1^{H362A}* mice. **b-c** RT-qPCR was performed to determine the expression levels of *Cxcl10* and *Tnfa* in indicated organs of *Enpp1^{WT}* and *Enpp1^{H362A}* mice. Cytokine transcript levels were normalized to the average of 2 uninfected controls per genotype. **d-h** Mice were inoculated with 2.5 x 10^7 PFU/mouse HSV-1 through intravenous injection. The mice were euthanized at 6 days post infection (dpi) and organs were isolated for total RNA isolation. *n* = 6 infected *Enpp1^{WT}* mice and *n* = 5 *ENPP1^{H362A}* mice. One lung *ENPP1^{H362A}* sample was excluded from **d-h** as an outlier based on the ROUT method (Q = 1%). *n.d.* = not detected. Data are shown as the mean ± SD. *p* values were calculated using the non-parametric Mann-Whitney test. *$p < 0.05$, **$p < 0.01$, ***$p < 0.001$; p value is shown if between 0.05 and 0.1.

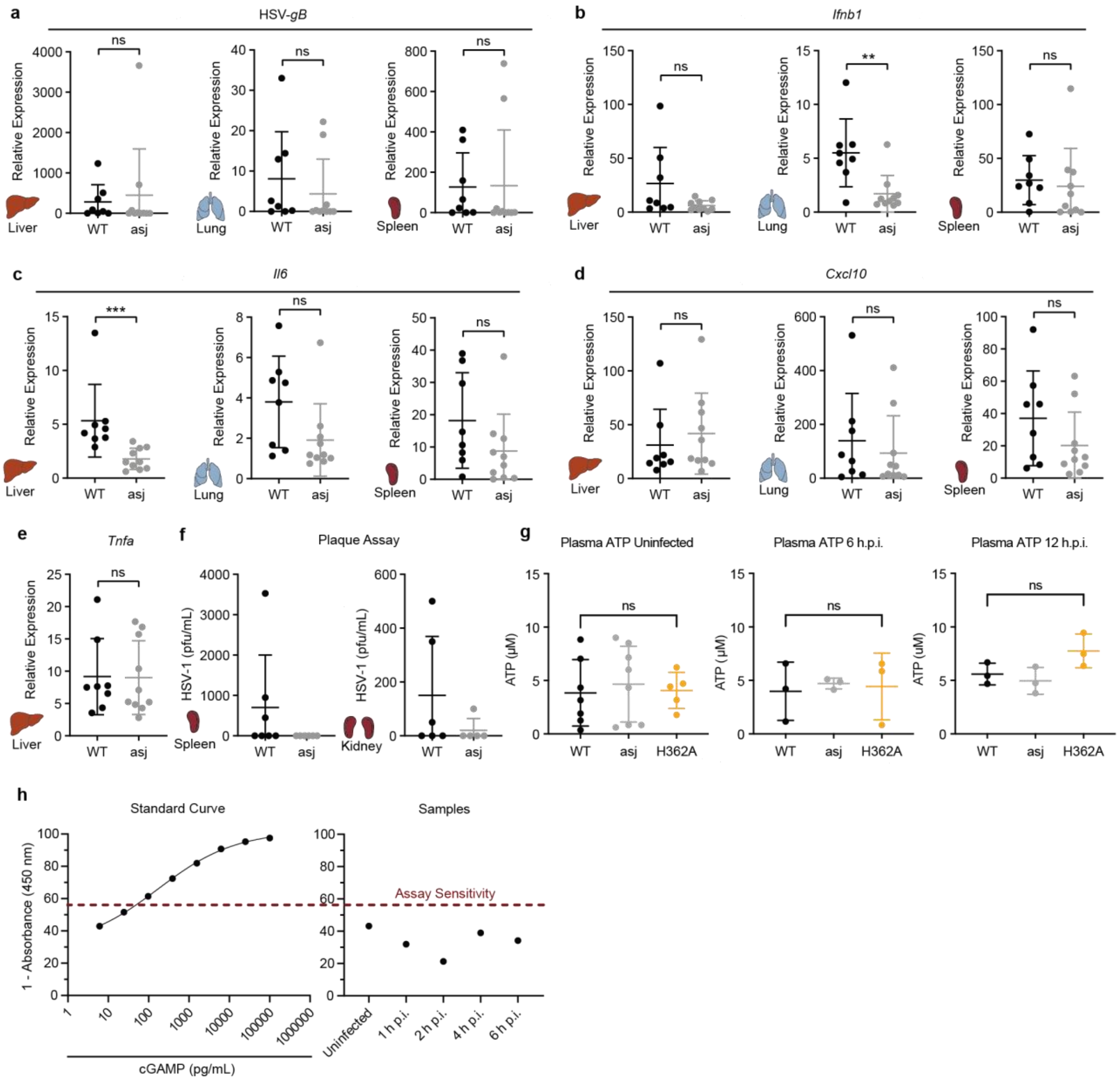

Supplementary Fig. 7 HSV-1 infection of *Enpp1*^{asj} and *Enpp1*^{H362A} mice. a-f Mice were inoculated with 2.5 x 10^7 PFU/mouse HSV-1 through intravenous injection. The mice were euthanized at 12 hpi and organs were isolated for plaque assays or total RNA isolation. *n* = 8 infected *Enpp1*^{WT} mice (replotted from Fig. 5) and *n* = 10 infected *ENPP1*^{asj} mice. RT-qPCR was performed to measure expression of HSV-*gB* (**a**), *Ifnb1* (**b**), *Il6* (**c**), *Cxcl10* (**d**), and *Tnfa* (**e**). Cytokine transcript levels were normalized to the average of 2 uninfected controls per genotype. **f** Plaque assays of spleen and kidney lysates from *Enpp1*^{WT} and *Enpp1*^{asj} mice. **g** Plasma ATP was measured from uninfected mice or mice infected with 2.5 x 10^7 PFU/mouse HSV-1 through intravenous injection. Plasma was collected from infected mice at 6 and 12 hpi. **h** *Enpp1*^{H362A} mice were injected with 2.5 x 10^7 PFU/mouse HSV-1 and euthanized at the indicated time points. Plasma was collected from each mouse, and cGAMP concentration was determined by cGAMP ELISA. A cGAMP standard curve was created in 50% mouse plasma (left). None of the infected plasma samples (right) gave readings above the published limit of detection (85 pg/mL), suggesting the absence of any cGAMP. For RT-qPCR data, transcript levels were normalized to the average of 2 uninfected mice per genotype. Data are shown as the mean ±

SD. p values were calculated using the Mann-Whitney test. *$p < 0.05$, **$p < 0.01$; p value is shown if between 0.05 and 0.1.

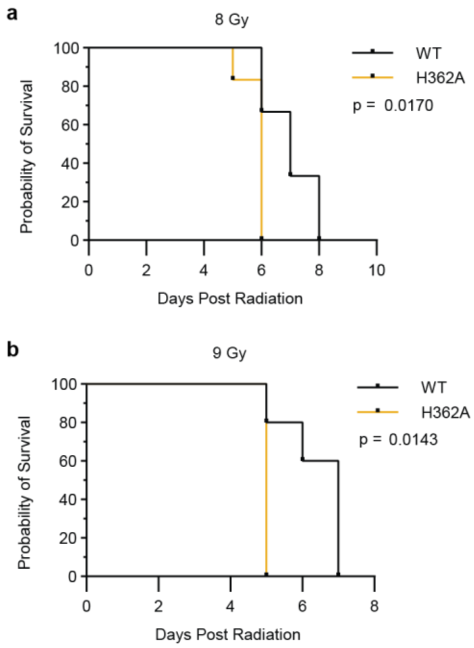

Supplementary Fig. 8. Enhanced extracellular cGAMP exacerbates radiation-induced inflammation. a-b Kaplan-Meier plots showing the probability of survival of the mice presented in Fig. 7, separated into mice receiving 8 Gy (**a**) and 9 Gy (**b**). n = 6 mice per genotype (**a**) or 5 mice per genotype (**b**). p value was calculated using a log rank (Mantel-Cox) test.

Supplementary Appendix 1. Detailed Methods

1. Synthesis and purification of cGAMP and [^{32}P]-cGAMP

To enzymatically synthesize cGAMP, 1 µM purified sscGAS was incubated with 20 mM Tris-HCl pH 7.4, 2 mM ATP, 2 mM GTP, 20 mM $MgCl_2$, and 100 µg/mL herring testis DNA (Sigma) for 24 h. The reaction was then heated at 95°C for 3 min and filtered through a 3-kDa filter. cGAMP was purified from the reaction mixture using a PLRP-S polymeric reversed phase preparatory column (100 Å, 8 µm, 300 x 25 mm; Agilent Technologies) on a preparatory HPLC (1260 Infinity LC system; Agilent Technologies) connected to UV-vis detector (ProStar; Agilent Technologies) and fraction collector (440-LC; Agilent Technologies). The flow rate was set to 25 mL/min. The mobile phase consisted of 10 mM triethylammonium acetate in water and acetonitrile. The mobile phase started as 2% acetonitrile for the first 5 min. Acetonitrile was then ramped up to 30% from 5-20 min, then to 90% from 20-22 min, maintained at 90% from 22-25 min, and then ramped down to 2% from 25-28 min. Fractions containing cGAMP were lyophilized and resuspended in water. The concentration was determined by measuring absorbance at 280 nm. To enzymatically synthesize [^{32}P]-cGAMP, 1 µM purified sscGAS was incubated with 20 mM Tris-HCl pH 7.4, 250 µCi (3000 Ci/mmol) [α-^{32}P]-ATP (Perkin Elmer), 1 mM GTP, 20 mM $MgCl_2$, and 100 µg/mL herring testis DNA (Sigma) in a reaction volume of 100 µL for 24 h. The reaction was purified by preparatory TLC on a HP-TLC silica gel plate (Millipore), eluted in water, and filtered through a 3-kDa filter to remove silica gel.

2. Enzyme activity assays

a) **cGAMP** activity assays (20 µL total) were composed of the following: cell/organ lysate (50%) or recombinant ENPP1 (1 – 10 nM), cGAMP (1 to 5 µM, with trace [^{32}P]-cGAMP spiked in), and buffer (standard assay buffer unless otherwise noted was 100 mM Tris pH 9 or pH 7.5, 150 mM NaCl, 500 µM $CaCl_2$, 10 µM $ZnCl_2$). At indicated times, 1 µL

aliquots of the reaction were quenched by spotting on HP-TLC silica gel plates (Millipore). The TLC plates were run in mobile phase (85% ethanol, 5 mM NH_4HCO_3) and exposed to a phosphor screen (GE BAS-IP MS). Screens were imaged on a Typhoon 9400 scanner.

b) **ATP** activity assays (10 μL total in a 384 well PCR plate) were composed of the following: cell/organ lysate (0.1–1%, depending on ENPP1 expression level) or recombinant ENPP1 (1 – 3 nM), 1 μM ATP (Sigma) and buffer (standard assay buffer unless otherwise noted was 100 mM Tris pH 9 or pH 7.6, 150 mM NaCl, 500 μM $CaCl_2$, 10 μM $ZnCl_2$). Reactions were started at indicated times and ended simultaneously by heating at 95 °C for 10 minutes. Reactions (5 μL) were transferred to a white 384 well plate, mixed with CellTiterGlo (5 μL), and luminescence was read after 15 minutes on a Tecan Spark plate reader.

c) **GTP, CTP, and UTP** activity assays were monitored by coupling pyrophosphate production to ATP production. To convert pyrophosphate into ATP, 5 μL of each plasma sample was added to a reaction mixture consisting of 16 μM adenosine phosphosulfate, 80 mM $MgSO_4$, 50 mM HEPES, and 0.1 μL ATP sulfurylase (MCLab). The reaction mixture was then incubated at 37°C for 10 min followed by 90°C for 10 min to inactivate the enzyme. In order to measure ATP, 25 μL of the reaction mixture was added to 25 μL of CellTiter-Glo (Promega). Luminescence was measured after 10 min using a 0.5 s integration time. For measurement of k_{cat}/K_m for recombinant ENPP1 with the substrate ATP, the commercial AMPGlo kit (Promega) was used according to the manufacturer's instructions.

d) **Additional cyclic dinucleotide** assays were composed of 100 nM *Xac* NPP, 25 μM cyclic dinucleotide, in a buffer consisting of 100 mM Tris pH 8, 150 mM NaCl, 100 μM $ZnCl_2$ (40 μL total). Reaction progress was measured by coupling the reaction to

alkaline phosphatase (1 U/µL of FastAP (ThermoFisher) per 40 µL reaction) and then measuring phosphate production with malachite green (Millipore Sigma).

3. Recombinant mouse ENPP1 purification

Procedures for culturing and transfecting Expi293F cells (Thermo Fisher) were based on the manufacturer's instructions. One day prior to transfection, the cells were split to 3×10^6 cells/mL in baffled flasks (Corning). On the day of transfection, cells were diluted to 3-4 $\times10^6$ cells/mL (if not already within the range) and transfected with plasmid DNA (0.5 µg DNA/mL cells) using FectoPro (Polyplus) (1 µL FectoPro/mL cells). Cells were immediately boosted with valproic acid (3 µM) and D-glucose (4 g/L). Cells were cultured for an additional 3 days. The media was harvested by centrifuging at 1000 x *g* for 10 minutes and passing through a 0.45 µm filter. Media was batch bound with HisPur cobalt resin (Thermo Fisher) (1 mL resin/ 60 mL culture) for 1 hour at 4 °C then loaded onto a fritted column. The column was washed two times with 2 column volumes (CV) wash buffer 1 (20 mM Tris pH 8.0, 150 mM NaCl, 10 mM imidazole) and once with 2 CV wash buffer 2 (20 mM Tris pH 8.0, 150 mM NaCl, 20 mM imidazole). Four elutions were performed with 1 CV elution buffer (20 mM Tris pH 8.0, 150 mM NaCl, 300 mM imidazole). Elution fractions were pooled and dialyzed against dialysis buffer (20 mM Tris pH 7.4, 150 mM NaCl) overnight at 4 °C. Protein was concentrated to 1 mg/mL in 20 mM Tris pH 7.4, 150 mM NaCl, 10% glycerol, and snap frozen for storage at -80 °C. Yield was ~1 mg ENPP1 per 60 mL of Expi293F culture.

4. Preparing ENPP1-transfected cell lysates

Plasmids containing Flag-ENPP1 WT or ENPP1 mutations were transfected into 293T *ENPP1*$^{-/-}$ cells with polyethylenimine (PEI) at ratio of 1 µg plasmid to 3 µg PEI per well of a 12 well plate. After 24 hours, cells were lysed for western blotting and activity assays. For western blotting,

cells were lysed on the plate in 150 μL of Laemmli sample buffer. For activity assays, cells were washed off the plate in 1 mL of PBS, centrifuged at 1000 x *g* for 10 minutes, and lysed in 100 μL of lysis buffer (10 mM Tris pH 9, 150 mM NaCl, 10 μM $ZnCl_2$, 1% NP-40). Lysates were stored at -20 °C.

5. Recombinant *Xac* NPP purification

Xac His-SUMO-NPP was expressed in BL21(DE3) cells. 1 L of bacteria grown in 2XYT media were induced at OD_{600} = 1 with 1 mM IPTG and grown at 16 °C for 16 hours. Bacteria were pelleted at 4000 x g and resuspended in column buffer (20 mM Tris pH 8, 150 mM NaCl, 20 μM $ZnCl_2$, 20 mM imidazole, and cOmplete protease inhibitor cocktail tablet (Sigma)). After two freeze-thaw cycles in liquid nitrogen, lysate was sonicated and centrifuged at 40,000 x *g* for 45 minutes. The supernatant was incubated with 1 mL of HisPur cobalt resin (ThermoFisher) for 1 hour at 4 °C, then washed with wash buffer (20 mM Tris pH 8, 150 mM NaCl, 20 μM $ZnCl_2$, 20 mM imidazole) and eluted with elution buffer (20 mM Tris pH 8, 150 mM NaCl, 20 μM $ZnCl_2$, 250 mM imidazole). The protein was dialyzed against 20 mM Tris pH 8 and incubated with protease Ulp1 (1:200 molar ratio) to cleave the His-SUMO tag overnight at 4 °C. Further purification was performed using anion exchange chromatography on a 1 mL HiTrap Q HP column (Cytiva) run on an Äkta Pure system (Cytiva). Buffer A = 20 mM Tris pH 8; Buffer B = 20 mM Tris pH 8, 1 M NaCl with a 20 CV ramp from zero NaCl to 1 M NaCl. Fractions of *Xac* NPP were pooled and dialyzed overnight at 4 °C against 20 mM Tris pH 8, 150 mM NaCl, 100 μM $ZnCl_2$, then stored at 4 °C until further use. The same procedures were used to purify *Xac* NPP^{WT}, *Xac* NPP^{T90A}, and *Xac* NPP^{H214A}.

6. Crystallization of *Xac* NPP^{T90A} with pApG and *Xac* NPP^{H214A} apo

Crystals of *Xac* NPP were grown at 20 °C by the hanging-drop vapor-diffusion method by mixing 1 μL of the protein solution (10 mg mL^{-1} in 5 mM Tris·HCl (pH 8.0), 150 mM NaCl, and 0.2 mM $ZnSO_4$), 1 μL of the reservoir solution (19% (vol/vol) PEG 3,350, 0.1 M Bis-tris, pH 6.0), and 10 mM cGAMP. *Xac* NPP^{T90A} crystals were harvested and soaked in mother liquor supplied with 1-10 mM of cGAMP and cryoprotectant right before cryo-cooling in liquid N_2. X-ray diffraction data were collected at beamline SSRL 12-2 (1) of Stanford Synchrotron Radiation Lightsource (SSRL) at SLAC National Accelerator Laboratory (Menlo Park, CA, USA) at cryogenic temperature using a single wavelength.

All diffracted *Xac* NPP^{T90A} crystals were in the $P2_12_12_1$ orthorhombic space group as has been reported previously (2). Regardless of cGAMP co-crystallization and/or varying soaking regimes, these orthorhombic crystals were bound to AMP rather than cGAMP or GMP. However, one new *Xac* NPP^{T90A} crystal form belonging to the monoclinic space group $P2_1$ was observed and diffracted to a minimum d-spacing of 1.90 Å. This monoclinic crystal contained six independent polypeptide chains in the asymmetry unit. The molecular replacement method with a previously published structure (PDB 2GSU) with all ligands stripped from the coordinate file was used as the search model with Phaser (3). The six copies of *Xac* NPP^{T90A} in the asymmetric unit of the $P2_1$ crystal could unambiguously be traced (chain A: His46-Met425; B: Thr44-Pro427; C: Ala42-Pro426; D: Thr44-Pro426; E: Thr44-Pro427; and F: Ser43-Pro426). The zinc ions were added in the bimetallic coordination sites provided by two triads: Asp54, Asp257, and His258; and Asp210, His214, and His363. Presence of extra electron density was evident after structure solution and accounted for AMP (five copies each one bound to the five polypeptide chains B-F) and one copy of partially hydrolyzed cGAMP (pApG). This partially hydrolyzed molecule was arbitrarily designated as chain A. Refinement of the structure with REFMAC (4) was done iteratively with visual inspection of electron density maps and manual adjustment of atomic coordinates in COOT (5) until progression to convergence. Careful inspection of electron density around the AMP positions revealed weak traces of density of what would correspond to

the GMP molecule. However, when atomic coordinates were built to these positions, the structure R_{factor}/R_{free} values did not improve and the modelled GMP had substantially higher B-factors (compared to the overall B-factor and atomic B-factors from neighboring residues). Therefore, GMP was not included in chains B-F. Solvent water molecules were manually assigned based on their hydrogen bonding properties. Refinement progressed to convergence and reached an excellent agreement between the model and the experimental data.

Apo *Xac* NPP^{H214A} crystals belonged to the $P2_12_12_1$ orthorhombic space group and diffracted to a d-spacing of 2.0 Å. Molecular replacement with 2GSU as the search model was as above. Apo *Xac* NPP^{H214A} is a mono-zinc structure as one of the two metal coordination sites is lost while the remaining site (provided by Asp54, Asp257, and His258) shows similar features as *Xac* NPP^{T90A}. In *Xac* NPP^{H214A} extra electron density accounted for two copies of polyethylene glycol (PEG 200) which presumably permeated the crystal from the cryoprotectant solution.

Supplementary Table 1 presents data collection, refinement, and structure quality check parameters. **Supplementary Table 2** presents ligand-protein interactions. Data was reduced with XDS (6), scaled with SCALA (7), and analyzed with different computing modules within the CCP4 suite (8). Graphic renderings were prepared with PyMOL (9). The final refined structure shows an excellent agreement with reference protein data as shown by Ramachandran statistics as surveyed with Molprobity (10). The structure has been validated and deposited with the RCSB Protein Data Bank (11) with codes 7MW8 (*Xac* NPP^{T90A} with pApG) and 7N1S (*Xac* NPP^{H214A} apo).

7. Computational modeling of substrates

A monomer of wild-type cGAMP-bound mENPP1 (PDB: 6AEK) was isolated and mutated on Pymol to generate $mENPP1^{H362A}$. The molecular coordinates of adenosine, including the alpha phosphate, were isolated together with Zn1009 and N-epsilon of H362, which was replaced by a

gamma phosphate oxygen to constitute an ATP structure with the free oxygens of both beta and gamma phosphates bonded to Zn resembling an approximately octahedral geometry, similar to previously proposed coordination between ATP and another 2+ ion, Mg^{2+} (12). The ligand was reassigned and used to replace cGAMP and Zn1009 on the mENPP1^{H362A} structure on Pymol. The composite structure was then minimized in Schrödinger Maestro using standard protein preparation workflow using all default settings at pH 7.4, with missing side chains filled against the corresponding FASTA sequence and using the OPLS3e force field. Similar minimization procedures were performed on *Xac* NPP^{H214A}, derived from the wild-type, pApG-bound structure (PDB: 7MW8, this paper). The bound pApG was used to prepare a receptor grid ($10\times10\times10$ Å^3), which was used to dock (using Glide at standard precision) all possible conformations of 2'3'-cGAMP and 3'3'-cGAMP, generated at pH 7.4 using LigPrep.

8. Generation and characterization of the transgenic *Enpp1^{H362A}* mouse strain

a) Generation. First, single-guide RNAs (sgRNAs) were designed against the H362 locus in exon 9 of mouse *Enpp1* using publicly available design tools (13) (**Supplementary Table 3**). The sgRNA was then complexed with Alt-R S.p. Cas9 nuclease (Integrated DNA Technologies) as a ribonucleoprotein (RNP) particle. We then designed a donor sequence based on mouse *Enpp1* to serve as the template for homologous recombination (**Supplementary Table 3**). The 100 nucleotide-long donor sequence contained blocking mutations near the PAM sequence to prevent repeated editing (14). The donor sequence was then synthesized as single-stranded DNA (Integrated DNA Technologies). The RNP particles and donor template were microinjected into the pronuclei of one-cell embryos from C57BL/6 mice, which were then implanted into pseudopregnant mice. As the initial litter of mice likely consisted of chimeras, the F1 generation was crossed with each other to generate a non-chimeric F2 generation. The

F2 generation was then sequenced, confirming the presence of homozygous *Enpp1*^{H362A} mutations in several mice.

b) **cGAMP ELISA for basal cGAMP detection**. Mice were euthanized and the spleens, kidneys, livers, and lungs were harvested. The spleens were diluted with 7.5 mL/g PBS and all other tissues were diluted with 2.5 mL/g PBS. The tissues were then homogenized and spun down at 2,000 x *g* for 15 min. A commercial cGAMP ELISA (Cayman Chemical) was used to determine the cGAMP concentration in each sample following the manufacturer's specifications.

c) **Histology sectioning and staining**. Organs were harvested at 20 weeks of age and fixed in 4% buffered Formaldehyde solution (pH 6.9) for 72 hours before transfer into 70% ethanol. Samples were submitted to Stanford Animal Histology Services for paraffin embedding, cutting and Alizarin Red staining. Imaging was done on a Zeiss AxioImager microscope in the Stanford Cell Sciences Imaging Facility.

d) **Plasma chemistry**. Blood was collected through terminal cardiac puncture into heparin-coated microtainers (BD). The blood was then spun at 2,000 x g for 15 min and the resulting plasma layer was collected. Plasma phosphate was measured using a malachite green phosphate assay kit (Sigma-Aldrich) according to the manufacturer's instructions; each sample was diluted 1:250 in water. Plasma calcium was measured using a commercial colorimetric assay (Stanbio) according to the manufacturer's instructions; each sample was diluted 1:4 in water. Plasma pyrophosphate was measured using a previously published method (15). To convert pyrophosphate into ATP, 5 µL of each plasma sample was added to a reaction mixture consisting of 16 µM adenosine phosphosulfate, 80 mM $MgSO_4$, 50 mM HEPEs, and 0.5 µL ATP sulfurylase (MCLab). The reaction mixture was then incubated at 37°C for 10 min, followed by 90°C for 10 min to inactivate the enzyme. In order to measure ATP, 25 µL of the

reaction mixture was added to 25 µL of CellTiter-Glo (Promega). Luminescence was measured after 10 min using a 0.5 s integration time.

e) ***In vivo* cGAMP metabolism.** Mice were injected subcutaneously with 5 mg/kg cGAMP diluted in 100 µL PBS. After 30 min, the mice were anesthetized with isoflurane and 50 µL of blood was collected retro-orbitally and immediately supplemented with ~20 µM of ENPP1 inhibitor STF-1623 to prevent cGAMP degradation. The blood was placed in heparin-coated microtainers (BD) and spun at 2,000 x g for 15 min and the resulting plasma layer was collected. The plasma was processed for LC-MS/MS by mixing plasma (7 µL) with acetonitrile containing 2 µM of internal standard cyclic GMP-$[^{13}C_{10},^{15}N_5]$AMP (20 µL), centrifuging at 16,000 x *g* for 15 min, and then adding 23 µL of the mixture to 15 µL of water containing 0.1% formic acid. cGAMP was analyzed on a Q-Exactive FT mass spectrometer (Thermo) equipped with a Vanquish UHPLC. Samples were injected onto a Biobasic AX LC column (5 µm, 50 × 3 mm; Thermo Scientific). The mobile phase consisted of 100 mM ammonium carbonate (A) and 0.1% formic acid in acetonitrile (B). The initial condition was 90% B, maintained for 0.5 min. The mobile phase was ramped to 30% A from 0.5 min to 2.0 min, maintained at 30% A from 2.0 min to 3.5 min, ramped to 90% B from 3.5 min to 3.6 min, and maintained at 90% B from 3.6 min to 5 min. The flow rate was set to 0.6 mL min^{-1}. Quantification was performed with TraceFinder 4.1 software (Thermo Fisher).

9. HSV-1 purification

The HSV-1 KOS strain was purchased from ATCC. The day prior to infection, Vero cells were plated in five T175 tissue culture flasks (Corning) at a density of 8 x 10^6 cells/flask so they would be 80-100% confluent on the day of infection. Cells were infected with HSV-1 at MOI 0.01 in 5 mL/flask of serum-free DMEM for 1 hour with gentle rocking every 15 minutes. Media was

collected 48 hours post infection when the cells displayed >90% CPE and centrifuged at 600 x *g* for 10 min to pellet debris. Clarified media was then centrifuged at 48,000 x *g* for 30 min to pellet virus. The pelleted virus was gently washed with PBS and resuspended in 2 mL of PBS, aliquoted, snap frozen, and stored at -80 °C until further use. Plaque assay was performed to determine titer (usually ~1 x 10^9 pfu/mL).

10. *In vivo* and *in vitro* HSV-1 infection models

2.5 x 10^7 PFU of HSV-1 (see **Supplementary Appendix 1** for purification procedures) was diluted in 100 μL PBS and injected intravenously into the tail vein of 6-9-week-old mice. After 6 h, 12 h, or 6 d the mice were euthanized in a CO_2 chamber and blood and organs were harvested. The blood was collected through cardiac puncture into heparin-coated microtainers (BD). The blood was then spun at 2,000 x g for 15 min, and the resulting plasma layer was collected. The organs were placed into collection tubes and frozen at -80°C until further processing, including plaque assays for determining infectivity, RT-qPCR for determining gene expression levels, and cGAMP measurement. Primary mouse bone marrow-derived macrophages (BMDMs) were infected with HSV-1 at indicated MOI and harvested at indicated time points. Gene expression was measured by RT-qPCR. See **Supplementary Appendix 1** for detailed methods.

11. HSV-1 plaque assays

One day prior to infection, Vero cells were plated at 0.2 x 10^6 cells/well in a 12-well plate or 0.1 x 10^6 cells/well in a 24-well plate so they would be 80-100% confluent on the day of infection. For tittering of HSV-1 stocks, 10-fold dilutions of HSV-1 were prepared in serum-free DMEM. For tittering of HSV-1 from tissues, previously frozen tissues were homogenized with 2.0 mm disruption beads (RPI) in Sarstedt tubes (Fisher, 50-809-242) using a tissue homogenizer. Final concentration of tissue homogenates was 500 mg/mL in PBS or serum-free DMEM. Tissue

homogenates were centrifuged at 500 x *g* for 5 minutes, and 2-fold and 20-fold dilutions were prepared in serum-free DMEM. For infection, Vero cells were washed one time with PBS, infected with 100 μL of sample for 1 hour with gentle rocking every 15 minutes, and then overlaid with complete DMEM containing 10 μg/mL human IgG (Sigma). 48 hours post infection, cells were fixed in 10% paraformaldehyde and stained with 0.4% crystal violet in 20% methanol.

12. Mouse BMDM isolation and infection

The bone marrow from mouse hind limb femurs and tibias was flushed by removing the end cap of the bones and centrifuging (16). Red blood cells were lysed by resuspending the pellet in red cell lysis buffer (155 mM NH_4Cl, 12 mM $NaHCO_3$, and 0.1 mM EDTA) and incubating for 5 min at room temperature. The cells were diluted in PBS, pelleted by centrifuge, and plated on one 10-cm dish per mouse in DMEM supplemented with 10% FBS, 1% P/S, and 10% conditioned L929 media. Cells were washed after 24 hours and allowed to differentiate for 5-7 days. To prepare for infection assay, BMDMs were plated at 80-90% confluence in 12-well plates. They were infected with various MOI of HSV-1 in 100 µL for 1 hour with periodic shaking. Virus-containing media was aspirated and replaced with fresh media.

13. RT-qPCR

Total RNA was isolated from cells and tissues using TRIzol (Invitrogen) by following the manufacturer’s protocol. Tissue samples were homogenized in TRIzol prior to RNA isolation. To obtain cDNA, 20 uL reverse transcriptase (RT) reactions were set up containing 1 µg total RNA, 100 pmol random hexamer primers, 0.5 mM dNTPs, 20 U RNaseOUT, 1x Maxima RT buffer, and 200 U Maxima RT (Thermo Scientific). RT reactions were incubated for 10 min at 25 °C, 15 min at 50 °C, then 5 min at 85 °C. To measure transcript levels, 10 µL qPCR reactions were set up containing 0.7 µL cDNA, 100 nM qPCR primers (**Supplementary Table 3**), and 1x AccuPower

GreenStar master mix (Bioneer) or 1x PowerTrack SYBR Green master mix (Thermo Scientific). Reactions were run on a ViiA 7 Real-Time PCR System (Applied Biosystems) using the following program: ramp up to 50°C (1.6°C/s) and incubate for 2 min, ramp up to 95°C (1.6°C/s) and incubate for 10 min, then 40 cycles of the following: ramp up to 95°C (1.6°C/s) and incubate for 15 s, then ramp down to 60°C (1.6°C/s) and incubate for 1 min. Transcript levels for each gene were normalized to *Actb* transcript levels.

14. cGAMP ELISA for plasma cGAMP measurement

2.5 x 10^7 PFU of HSV-1 was diluted in 100 μL PBS and injected intravenously into the tail vein of each mouse. After the indicated timepoints, the mice were euthanized in a CO_2 chamber and the blood was collected through cardiac puncture into heparin-coated microtainers (BD). The blood was then spun at 2,000 x g for 15 min and the resulting plasma layer was collected. A commercial cGAMP ELISA (Cayman Chemical) was used to determine the cGAMP concentration in each sample following the manufacturer's specifications. Each sample was diluted 1:2 in the provided buffer and the standard curve was generated in buffer mixed with 50% mouse plasma from uninfected *Enpp1*^{H362A} mice.

15. Total body irradiation mouse model

Male and female 8-12-week-old mice were irradiated with either 8 or 9 Gy using a 225 kVp cabinet X-ray irradiator with a 0.5 mm Cu filter (IC-250, Kimtron Inc.). Mice were anesthetized with a mixture of 80 mg/kg ketamine (VetaKet) and 5 mg/kg xylazine (AnaSed) prior to irradiation. The mice were weighed daily and were euthanized if they met the humane endpoint of greater than 20% weight loss for two consecutive days. 50 µL of blood was withdrawn retro-orbitally 5 days after irradiation for IFN-β ELISA analysis. The blood was spun at 2,000 x g for 15 min and the resulting plasma layer was collected. A commercial high-sensitivity IFN-β ELISA kit (PBL Assay Science) was used to determine the IFN-β concentration in each sample

following the manufacturer’s specifications. Each sample was diluted 1:10 in the provided buffer and the standard curve was generated in buffer mixed with 10% mouse plasma from healthy mice. Finally, spleens were harvested at endpoint for RT-qPCR analysis.

Supplementary Table 1. Crystallographic data collection and refinement statistics

	T90A Xac NPP/pApG - AMP	H214A Xac NPP
**Data collection**		
Beamline	SSRL BL12-2	SSRL BL12-2
Wavelength (Å)	1.18076	0.97946
Space group	$P2_1$	$P2_1 2_1 2_1$
Cell dimensions		
a, *b*, *c* (Å)	130.42, 66.72, 134.96	65.20, 77.71, 129.29
α, β, γ (°)	90.00, 116.25, 90.00	90.00, 90.00, 90.00
Matthews coefficient ($Å^3$/Da)[a]	2.03	1.92
Solvent content (%)	39.3	38.3
Wilson B value ($Å^2$)	26.6	30.6
Anisotropy	0.76	0.68
Resolution (Å)[b]	39.0(1.9)	38.9(2.0)
R_{merge}[c]	0.132(0.596)	0.101(1.192)
$I/\sigma I$ ratio[d]	3.7(1.1)	9.7(1.8)
Completeness (%)[e]	95.0(89.1)	100(100)
Reflections (total/unique)	449,161(155,855)	328,450(45,218)
Redundancy[f]	2.9(2.6)	7.3(7.2)
**Refinement**		
Resolution (Å)	30.00-1.90	30.61-2.00
No. reflections/test set	147,590/7,565	45,168/2,185
R_{work} / R_{free}[g]	24.3/30.0	20.9/26.2
Mean B value ($Å^2$)	37.0	44.3
F_{obs}-F_{calc} correlation[h]	0.94	0.96
No. atoms		
Protein	17,617	5,845
Ligand/ion	173 (1 pApG/5 AMP/12 Zn^{2+})	28 (2 Zn^{2+}/2 PEG)
Water	386	99
B-factors		
Protein	38.3 (chain A), 33.9 (B), 35.2 (C), 44.2 (D), 41.4 (E), 33.9 (F)	43.8 (chain A), 45.3 (B)
Ligand/ion	43 (pApG)/27-40 (AMP)/28.2 (Zn^{2+})	47.7 (Zn^{2+})/44.9 (PEG)
Water	33.3	42.2
Deviation from ideality (Rmsd values)	0.013 Å (bond length), 1.685° (bond angle)	0.014 Å (bond length), 1.711° (bond angle)
Ramachandran statistics[i]		
Most favored/allowed regions (%)	99.5 (2,277 over 2,292)	99.9 (757 over 761)
Disallowed regions (%)	0.5 (15 over 2,292)	0.1 (4 over 761)
PDB code	7MW8	7N1S

[a]Ratio of the volume of the asymmetric unit to the molecular weight of all protein in the asymmetric unit
[b]Value in parentheses is for the highest-resolution shell: 1.90 – 2.00 Å.

[c]Reliability factor for symmetry-related reflections calculated as: $R_{merge} = \Sigma_{hkl}\ \Sigma j{=}1 \text{ to } N \mid I_{hkl} - I_{hkl}(j) \mid / \Sigma_{hkl}\ \Sigma j{=}1 \text{ to } N\ I_{hkl}(j)$, where N is the redundancy of the data. In parentheses, the cumulative value at the highest-resolution shell
[d]Ratio of mean intensity to the mean standard deviation of the intensity over the entire resolution range
[e]Fraction of measured reflections to possible observations at the resolution range
[f]Number of measurements of individual, symmetry unique reflections
[g]Average deviation between the observed and calculated structure factors calculated as: $R_{work} = \Sigma_{hkl} ||F_{obs}| - |F_{calc}|| / \Sigma_{hkl} |F_{obs}|$, where the F_{obs} and F_{calc} are the observed and calculated structure factor amplitudes of reflection hkl. R_{free} is equal to R_{factor} but for a randomly selected 5.0 % subset of the total reflections that were held aside throughout refinement for cross-validation
[h]Correlation coefficient between observed and calculated structure factor amplitudes
[i]According to Molprobity for non-proline and non-glycine residues

Supplementary Table 2. Intermolecular contacts between pApG/AMP and *Xac* NPP^{T90A}

Xac NPP^{T90A}: A		Distance (Å)	Type of interaction: pApG
His214	Nδ1	3.43	H-bond O3' GMP
Asn111	Nδ2	2.72	H-bond O1P 5' PO4 group
Ala90	NH	2.94	H-bond O1P 5' PO4 group to main-chain NH
Lys176	Nζ	2.72<br>3.38	H-bonds O3' and O1P 3' PO4 group
Tyr174	Oη	2.69	H-bond O2' AMP
Ser155	Oγ	3.41	Water molecule H-bonded to N6 (2.92 Å) and N7 (2.57 Å) AMP
Phe91	Ring	avg. 3.7	T-shaped hydrophobic interaction with AMP
Zn2		2.19, 2.73	Coordination O2P, O3P 5' PO4
Tyr174	Ring	avg. 3.5	Stacking hydrophobic interaction with AMP
Zn1		1.88	Coordination O3P 5' PO4
Leu123	NH	3.08	H-bond O2 GMP
Ser112	Oγ	2.68<br>3.30	H-bonds N1, N6 GMP
Asp285	NH<br>Oγ1	3.14<br>3.46	Water molecule H-bonded to N6 GMP

Xac NPP^{T90A}: B-F		Distance (Å)	Type of interaction: AMP
Asn111	Nδ2	2.71	H-bond O1P 5' PO4 group
Ala90	NH	2.94	H-bond O1P 5' PO4 group to main-chain NH
Lys176	Nζ	2.72<br>3.38	H-bonds O3' and O1P 3' PO4 group
Tyr174	Oη	2.69	H-bond O2' AMP
Ser155	Oγ	3.41	Water molecule H-bonded to N6 (2.92 Å) and N7 (2.57 Å) AMP
Phe91	Ring	avg. 3.7	T-shaped hydrophobic interaction with AMP
Zn2		2.19, 2.73	Coordination O2P, O3P 5' PO4
Zn1		1.88	Coordination O3P 5' PO4
Tyr174	Ring	avg. 3.5	Stacking hydrophobic interaction with AMP

Supplementary Table 3. Oligonucleotides used in this study

**Primers for Cloning:**	
**Name**	**Sequence (5'->3')**
mENPP1_D200A_fwd	CCCCCTACTCTCTTGTTTTCTTTGGCTGGATTCAGAGCTG
mENPP1_D200A_rev	CAGCTCTGAATCCAGCCAAAGAAAACAAGAGAGTAGGGGG
mENPP1_K237A_fwd	CCTATGTACCCTACCGCAACGTTTCCCAATCATTACAGC
mENPP1_K237A_rev	GCTGTAATGATTGGGAAACGTTGCGGTAGGGTACATAGG
mENPP1_T238A_fwd	GCCTATGTACCCTACCAAGgcgTTTCCCAATCATTACAGC
mENPP1_T238A_rev	GCTGTAATGATTGGGAAAcgcCTTGGTAGGGTACATAGGC
mENPP1_N259A_fwd	CCCATGGCATAATTGATGCAAAGATGTATGATCCC
mENPP1_N259A_rev	GGGATCATACATCTTTGCATCAATTATGCCATGGG
mENPP1_K260A_fwd	CCCATGGCATAATTGATAACGCAATGTATGATCCCAAAATGAATGC
mENPP1_K260A_rev	GCATTCATTTTGGGATCATACATTGCGTTATCAATTATGCCATGGG
mENPP1_D358A_fwd	GTATTTAGAAGAACCAGcTTCTTCAGGGCATTCACATGG
mENPP1_D358A_rev	CCATGTGAATGCCCTGAAGAAgCTGGTTCTTCTAAATAC
mENPP1_H362A_fwd	CCAGATTCTTCAGGGGCATCACATGGACCAGTCAGC
mENPP1_H362A_rev	GCTGACTGGTCCATGTGATGCCCCTGAAGAATCTGG
mENPP1_D405A_fwd	CCTCATCCTCATTTCAGcTCACGGCATGGAACAAGG
mENPP1_D405A_rev	CCTTGTTCCATGCCGTGAgCTGAAATGAGGATGAGG
mENPP1_H406A_fwd	CATCCTCATTTCAGATgcCGGCATGGAACAAGGCAGC
mENPP1_H406A_rev	GCTGCCTTGTTCCATGCCGgcATCTGAAATGAGGATG
mENPP1_Y433A_fwd	GGATGTGAACAATGTGAAAGTTGTGGCAGGACCTGCTGCTCGG
mENPP1_Y433A_rev	CCGAGCAGCAGGTCCTGCCACAACTTTCACATTGTTCACATCC
mENPP1_P435A_fwd	GTGAAAGTTGTGTATGGAGCAGCTGCTCGGTTGAGACCC
mENPP1_P435A_rev	GGGTCTCAACCGAGCAGCTGCTCCATACACAACTTTCAC
mENPP1_Q501A_fwd	CCTGGACCCTCAGTGGGCACTTGCGTTGAATCCATCAGAGAGG
mENPP1_Q501A_rev	CCTCTCTGATGGATTCAACGCAAGTGCCCACTGAGGGTCCAGG
mENPP1_K510A_fwd	CCATCAGAGAGGGCATATTGTGGAAGTGG
mENPP1_K510A_rev	CCACTTCCACAATATGCCCTCTCTGATGG
mENPP1_Y511A_fwd	CCATCAGAGAGGAAAGCATGTGGAAGTGGATTTCATGG
mENPP1_Y511A_rev	CCATGAAATCCACTTCCACATGCTTTCCTCTCTGATGG
mENPP1_S514A_fwd	GGAAATATTGTGGAGCAGGATTTCATGGCTCTGAC
mENPP1_S514A_rev	GTCAGAGCCATGAAATCCTGCTCCACAATATTTCC
mENPP1_F516A_fwd	GGAAATATTGTGGAAGTGGAGCACATGGCTCTGACAAC
mENPP1_F516A_rev	GTTGTCAGAGCCATGTGCTCCACTTCCACAATATTTCC
mENPP1_H517A_fwd	GTGGAAGTGGATTTgcTGGCTCTGACAACTTG
mENPP1_H517A_rev	CAAGTTGTCAGAGCCAgcAAATCCACTTCCAC
mENPP1_H362R_fwd	CCAGATTCTTCAGGGcgtTCACATGGACCAGTCAGC
mENPP1_H362R_rev	GCTGACTGGTCCATGTGAacgCCCTGAAGAATCTGG
mENPP1_H362E_fwd	CCAGATTCTTCAGGGgagTCACATGGACCAGTCAGC
mENPP1_H362E_rev	GCTGACTGGTCCATGTGActcCCCTGAAGAATCTGG
mENPP1_H362S_fwd	CCAGATTCTTCAGGGtctTCACATGGACCAGTCAGC
mENPP1_H362S_rev	GCTGACTGGTCCATGTGAagaCCCTGAAGAATCTGG

mENPP1_H362T_fwd	CCAGATTCTTCAGGGactTCACATGGACCAGTCAGC
mENPP1_H362T_rev	GCTGACTGGTCCATGTGAagtCCCTGAAGAATCTGG
mENPP1_H362N_fwd	CCAGATTCTTCAGGGaatTCACATGGACCAGTCAGC
mENPP1_H362N_rev	GCTGACTGGTCCATGTGAattCCCTGAAGAATCTGG
mENPP1_H362C_fwd	CCAGATTCTTCAGGGtgtTCACATGGACCAGTCAGC
mENPP1_H362C_rev	GCTGACTGGTCCATGTGAacaCCCTGAAGAATCTGG
mENPP1_H362G_fwd	CCAGATTCTTCAGGGggtTCACATGGACCAGTCAGC
mENPP1_H362G_rev	GCTGACTGGTCCATGTGAaccCCCTGAAGAATCTGG
mENPP1_H362P_fwd	CCAGATTCTTCAGGGcctTCACATGGACCAGTCAGC
mENPP1_H362P_rev	GCTGACTGGTCCATGTGAaggCCCTGAAGAATCTGG
mENPP1_H362I_fwd	CCAGATTCTTCAGGGattTCACATGGACCAGTCAGC
mENPP1_H362I_rev	GCTGACTGGTCCATGTGAaatCCCTGAAGAATCTGG
mENPP1_H362M_fwd	CCAGATTCTTCAGGGatgTCACATGGACCAGTCAGC
mENPP1_H362M_rev	GCTGACTGGTCCATGTGAcatCCCTGAAGAATCTGG
mENPP1_H362W_fwd	CCAGATTCTTCAGGGtggTCACATGGACCAGTCAGC
mENPP1_H362W_rev	GCTGACTGGTCCATGTGAccaCCCTGAAGAATCTGG
mENPP1_H362Y_fwd	CCAGATTCTTCAGGGtatTCACATGGACCAGTCAGC
mENPP1_H362Y_rev	GCTGACTGGTCCATGTGAataCCCTGAAGAATCTGG
mENPP1_H362V_fwd	CCAGATTCTTCAGGGgttTCACATGGACCAGTCAGC
mENPP1_H362V_rev	GCTGACTGGTCCATGTGAaacCCCTGAAGAATCTGG
mENPP1_seq_rev	CCATTATTGGGAGCTGGGATCAAACC
mENPP1_seq_fwd	CTACAGTTCTGTGTGCCAAG
XacNPP_H214A_fwd	CATGTGGACGAAGCCGGCgcCGACCACGGCCCGGAATCGC
XacNPP_H214A_rev	GCGATTCCGGGCCGTGGTCGgcGCCGGCTTCGTCCACATG
**Primers for qPCR:**	
**Name**	**Sequence (5'->3')**
Cxcl10 Fwd	AAGTGCTGCCGTCATTTTCT
Cxcl10 Rev	GTGGCAATGATCTCAACACG
Irf7 Fwd	GAAGACCCTGATCCTGGTGA
Irf7 Rev	CCAGGTCCATGAGGAAGTGT
HSV-gB Fwd	ATTCTCCTCCGACGCCATATCCACCACCTT
HSV-gB Rev	AGAAAGCCCCCATTGGCCAGGTAGT
Actb Fwd	AGCCATGTACGTAGCCATCC
Actb Rev	CTCTCAGCTGTGGTGGTGAA
**Oligonucleotides for generating the ENPP1^{H362A} mouse:**	
**Name**	**Sequence (5'->3')**
Enpp1 Exon 9 sgRNA	GATTCTTCAGGGCATTCACA
Enpp1 Exon 9 Sequencing Fwd	GATGATTTATAGCCAGAGCAACTAGTG
Enpp1 Exon 9 Sequencing Rev	GTTCTCTCTGGCTACATAGAATTTC
Enpp1 H362A Donor Sequence for Homologous Recombination	TGTTTTTCAATGTGTTCGTAAAATGTTACATTTTGATACTGTT TGATTTAGACCACACTTTTACACTCTGTATTTAGAAGAACCA GATTCTTCAGGGGCAAGTCATGGACCAGTCAGCAGCGAGG TAAGTTCACCGCTACCTATAATCACTTCGTTAAATTTAGTATT CCTGAAGTGGACTTCAAGAACCTTCTGTAAGG

Supplementary Table 4. Protein Accession Numbers for Representative Species

Species	Protein accession number
Mouse (*Mus musculus)*	P06802
Human (*Homo sapiens*)	P22413
Chicken (*Gallus gallus*)	XP_040523241.1
Frog (*Xenopus tropicalis*)	XP_031758349.1
Zebrafish (*Danio rerio*)	NP_001025339.1
Sea anemone (*Nematostella vectensis*)	XP_032232033.1
Rice (*Oryza sativa*)	XP_025879414.1
Thale cress (*Arabidopsis thaliana*)	NP_194697.1
Roundworm (*Caenorhabditis elegans*)	NP_001041086.1
Baker's yeast (*Saccharomyces cerevisiae*)	PTN13750.1
Bacteria (*Xanthomonas axonopodis pv. citri*)	Q8PIS1_XANAC
Bacteria (*Streptomyces lavendulae*)	ATZ26543.1
Bacteria (*Porphyromonas gingivalis*)	PDP83833.1
Bacteria (*Bacillus cereus*)	Q73E52_BACC1

References

1. Russi S, Song J, McPhillips SE, & Cohen AE (2016) The Stanford Automated Mounter: pushing the limits of sample exchange at the SSRL macromolecular crystallography beamlines. *J Appl Crystallogr* 49(Pt 2):622-626.
2. Zalatan JG, Fenn TD, Brunger AT, & Herschlag D (2006) Structural and functional comparisons of nucleotide pyrophosphatase/phosphodiesterase and alkaline phosphatase: implications for mechanism and evolution. *Biochemistry* 45(32):9788-9803.
3. McCoy AJ*, et al.* (2007) Phaser crystallographic software. *J Appl Crystallogr* 40(Pt 4):658-674.
4. Murshudov GN, Vagin AA, & Dodson EJ (1997) Refinement of macromolecular structures by the maximum-likelihood method. *Acta Crystallogr D Biol Crystallogr* 53(Pt 3):240-255.
5. Emsley P, Lohkamp B, Scott WG, & Cowtan K (2010) Features and development of Coot. *Acta Crystallogr D Biol Crystallogr* 66(Pt 4):486-501.
6. Kabsch W (2010) Xds. *Acta Crystallogr D Biol Crystallogr* 66(Pt 2):125-132.
7. Evans PR (2011) An introduction to data reduction: space-group determination, scaling and intensity statistics. *Acta Crystallogr D Biol Crystallogr* 67(Pt 4):282-292.
8. Winn MD*, et al.* (2011) Overview of the CCP4 suite and current developments. *Acta Crystallogr D Biol Crystallogr* 67(Pt 4):235-242.
9. DeLano WL (2002) Pymol: An open-source molecular graphics tool. *CCP4 Newsletter Pro. Crystallogr* 40:82-92.
10. Davis IW*, et al.* (2007) MolProbity: all-atom contacts and structure validation for proteins and nucleic acids. *Nucleic Acids Res* 35(Web Server issue):W375-383.
11. Berman HM*, et al.* (2002) The Protein Data Bank. *Acta Crystallogr D Biol Crystallogr* 58(Pt 6 No 1):899-907.

12. Dudev T, Grauffel C, & Lim C (2017) How Native and Alien Metal Cations Bind ATP: Implications for Lithium as a Therapeutic Agent. *Sci Rep* 7:42377.
13. Haeussler M*, et al.* (2016) Evaluation of off-target and on-target scoring algorithms and integration into the guide RNA selection tool CRISPOR. *Genome Biol* 17(1):148.
14. Okamoto S, Amaishi Y, Maki I, Enoki T, & Mineno J (2019) Highly efficient genome editing for single-base substitutions using optimized ssODNs with Cas9-RNPs. *Sci Rep* 9(1):4811.
15. Oheim R*, et al.* (2020) Human Heterozygous ENPP1 Deficiency Is Associated With Early Onset Osteoporosis, a Phenotype Recapitulated in a Mouse Model of Enpp1 Deficiency. *J Bone Miner Res* 35(3):528-539.
16. Heib T, Gross C, Muller ML, Stegner D, & Pleines I (2021) Isolation of murine bone marrow by centrifugation or flushing for the analysis of hematopoietic cells - a comparative study. *Platelets* 32(5):601-607.
